# Optimizing Parkinson’s disease progression scales using computational methods

**DOI:** 10.1038/s41531-026-01259-1

**Published:** 2026-01-23

**Authors:** Assaf Benesh, Roy N. Alcalay, Anat Mirelman, Ron Shamir

**Affiliations:** 1 Blavatnik School of Computer Science and Artificial Intelligence, Tel Aviv University, Tel Aviv, Israel; 2 Movement Disorders Division, Neurological Institute, Tel Aviv Sourasky Medical Center, Tel Aviv, Israel; 3 Department of Neurology, Columbia University Irving Medical Center, New York, NY USA; 4 Gray Faculty of Medicine and Health Sciences and Sagol School of Neuroscience, Tel Aviv University, Tel Aviv, Israel; 5 Laboratory for Early Markers of Neurodegeneration (LEMON), Neurological Institute, Tel Aviv Sourasky Medical Center, Tel Aviv, Israel

**Keywords:** Computational biology and bioinformatics, Diseases, Neurology, Neuroscience

## Abstract

Parkinson’s disease (PD) is a highly heterogeneous condition with symptoms spanning motor and non-motor domains. Clinical scales like the Movement Disorder Society’s Unified Parkinson’s Disease Rating Scale (MDS-UPDRS) are standard in clinical trials where disease progression is monitored. They rely on summing item values, assuming uniform item importance and score increments. Here, we propose a novel data-driven approach to optimize weights for such scales–so that total scores better reflect the underlying disease severity. In a retrospective observational analysis of longitudinal cohort data from the Parkinson’s Progression Markers Initiative (PPMI), our methods identified which items (and value increments) most strongly indicate PD progression, down-weighting or excluding less informative items. The learned weights substantially improve the monotonic relationship between total scores and clinical progression. We validated our weights using both held-out PPMI data and an independent dataset (BeaT-PD), demonstrating their robustness. Applying such weights in clinical trials may increase power and reduce the required sample size^1^.

## Introduction

Parkinson’s disease (PD) is a complex, progressive neurological disorder characterized by a range of motor and non-motor symptoms. The most commonly used assessment in PD is the Movement Disorder Society’s Unified Parkinson’s Disease Rating Scale (MDS-UPDRS)^2^, a 65-item scale divided into four parts. Each item has five possible answers numbered 0 to 4, reflecting increasing severity. Although thoroughly validated^3–5^ and widely accepted^6^, MDS-UPDRS exhibits several limitations. First, the total score is obtained by summing item scores, assuming that all items-and all increments within items-are equally informative. For instance, a score of 2 on two different items could have markedly different clinical implications, yet both add the same amount to the total score. Similarly, increasing an item’s score from 0 to 2 has the same effect on the total score as increasing it from 2 to 4, although these increments have different clinical significance. Second, administering the full scale is time-consuming and some items may not consistently contribute to tracking disease progression.

Due to these reasons, a more robust measure of disease trajectory is needed—one that captures underlying progression better. Moreover, identifying and discarding redundant or minimally informative questions can streamline patient evaluations, reducing both clinical burden and patient fatigue. This work aimed to optimize the weighting of MDS-UPDRS (and related) scale items in order to produce a more accurate and concise PD progression index, capture biology better, and help reduce recruitment needs for clinical trials.

We formulated an optimization problem that seeks weights yielding a score that increases as patients progress, thereby providing a more accurate representation of the true disease state, which we assume progresses monotonically (but not necessarily linearly^7^) over time^8–10^. To reflect this monotonicity we wish to optimize *consistency*—defined as the proportion of exam pairs for which the later exam attains a higher score than the earlier one. Note that only pairs of exams of the same patient are compared, and the proportion is calculated across all patients. Concretely, for each MDS-UPDRS question we allowed assigning different weights to the increments between answers (0 to 1, 1 to 2, 2 to 3, 3 to 4), and possibly different weights for different questions, and leveraged computational methods (e.g., linear or integer programming) to choose the weights such that the longitudinal monotonicity of the data is maximized. This data-driven approach enabled us to (1) discover the relative importance of different items and scores, (2) reduce the influence of medication-induced fluctuations, and (3) minimize redundancy by identifying and down-weighting less informative items.

We constructed six indexes corresponding to two main groups of formulations of optimization problems: MeanDiff-based variants, which aim to maximize the overall increase in scores between earlier and later examinations of the same patient, and Cons-based variants, which directly optimize consistency. Of the four MeanDiff variants, MeanDiff maximizes the mean score difference between visits; MeanDiff-W maximizes the mean weighted score, assigning a larger penalty for decreases than the reward to increases; MeanDiff-QP applies a quadratic penalty for decreases, and MeanDiff-SV encourages low variance in score difference. The two Cons variants are Cons, which directly optimizes consistency, and Cons-Int, which does so while enforcing integer weights.

We used data from the Parkinson’s Progression Markers Initiative (PPMI)^11^ to develop the indexes and validated them on held-out PPMI data (see participant selection flow diagram in Fig. S1 of the Supplementary Information), external progression criteria, and an external cohort of PD patients obtained from the BeaT-PD project (204-16TLV)^12^. The resulting indexes offer greater accuracy and more concise measurement instruments.

## Results

### Comparing the performance of the different approaches

Table 1 presents the characteristics of the final PPMI cohort after data cleaning.

**Table 1:** PPMI participant characteristics

Characteristic	Mean	Std	Min	Q1	Median	Q3	Max
Age (years)	63.29	9.6	33.2	56.8	64.4	70.4	85.9
Disease duration (years)	2.54	1.9	0.92	1.33	1.75	3.0	14.09
MDS-UPDRS total score	36.65	16.62	4	25	35	46	122
H&Y Stage	1.78	0.55	0	1	2	2	4
MoCA total score	26.22	3.25	6	24	27	28	30
Gender (M%)	61.6%						
Follow-up time (years)	4.78	3.23	0.5	1.92	4.08	7.0	12.08
Number of visits	4.63	2.47	2	2	4	6	12

Results are shown for PPMI cohort after filtering (3295 examinations for 711 PD patients). The first five characteristics listed correspond to each patient’s first visit included in the analysis. Q1: 25th percentile; Q3: 75th percentile.

*MDS-UPDRS* Movement Disorder Society’s Unified Parkinson’s Disease Rating Scale, *MoCA* Montreal Cognitive Assessment, *H&Y* Hoehn and Yahr.

Table 2 shows the results when all items were used, as well as the results from applying the original weights of the complete MDS-UPDRS, its individual sections, and MoCA. The new methods outperformed the MDS-UPDRS and its parts as well as MoCA, with MeanDiff-QP performing best.

**Table 2:** Performance comparison when all MDS-UPDRS and MoCA items are used

Years Gap	1	2	3	4	5	6	7	8	9	10	All
**Number of Pairs**	417	319	242	185	134	96	71	53	38	21	1576
**MDS-UPDRS P1**	49.64	56.43	55.79	64.32	62.69	68.75	70.42	73.58	73.68	76.19	58.63
**MDS-UPDRS P2**	53.00	58.31	60.33	69.19	72.39	78.12	83.10	90.57	92.11	95.24	64.40
**MDS-UPDRS P3**	50.84	54.55	47.93	51.89	55.97	54.17	69.01	75.47	81.58	80.95	54.70
**MDS-UPDRS**	54.44	57.05	57.44	63.24	66.42	63.54	78.87	84.91	89.47	90.48	61.48
**MoCA**	38.13	37.93	38.43	40.00	44.03	43.75	32.39	45.28	42.11	57.14	39.53
**MeanDiff**	55.40	57.68	61.57	69.73	73.88	76.04	83.10	84.91	92.11	85.71	64.85
**MeanDiff-W**	58.51	62.07	62.81	72.97	76.12	80.21	85.92	90.57	92.11	90.48	67.96
**MeanDiff-QP**	**62.35**	**66.14**	**73.14**	**78.38**	**85.07**	**91.67**	**91.55**	**98.11**	**97.37**	**100.00**	**74.24**
**MeanDiff-SV**	59.71	63.01	64.05	72.97	79.85	81.25	88.73	92.45	94.74	95.24	69.35
**Cons**	59.71	64.26	69.01	76.76	79.85	84.38	88.73	94.34	97.37	95.24	71.13
**Cons-Int**	58.27	65.52	68.18	76.76	**85.07**	85.42	**91.55**	88.68	94.74	**100.00**	71.32

The table shows the percentage of consistent pairs of visits for each method, for different time gaps between the visits. Time gaps are rounded to the closest year. The number in bold shows the best performer for each gap. The last column gives the weighted average percentage of consistent pairs.

*MDS-UPDRS* Movement Disorder Society’s Unified Parkinson’s Disease Rating Scale, *MoCA* Montreal Cognitive Assessment.

When limited to self-reported items (Table 3), a similar advantage of the new scale is observed. MeanDiff-QP performs on par with the Cons method, with the latter being slightly better for shorter time gaps.

**Table 3:** Performance comparison when only the self-reported items in MDS-UPDRS are used

Years Gap	1	2	3	4	5	6	7	8	9	10	All
**Number of Pairs**	417	319	242	185	134	96	71	53	38	21	1576
**MDS-UPDRS P1**	46.76	52.66	58.68	61.62	57.46	65.62	71.83	75.47	76.32	66.67	56.66
**MDS-UPDRS P2**	53.00	58.31	60.33	69.19	72.39	78.12	83.10	90.57	92.11	95.24	64.40
**MDS-UPDRS**	53.96	61.13	66.94	74.05	74.63	72.92	85.92	90.57	94.74	**100.00**	66.94
**MeanDiff**	54.44	61.76	66.94	72.43	76.12	73.96	84.51	92.45	94.74	**100.00**	67.20
**MeanDiff-W**	54.20	61.13	66.12	72.43	77.61	76.04	84.51	90.57	94.74	**100.00**	67.07
**MeanDiff-QP**	58.99	61.13	**71.90**	**76.22**	82.09	84.38	**91.55**	**96.23**	**97.37**	**100.00**	71.13
**MeanDiff-SV**	58.51	64.89	67.36	74.05	76.12	80.21	88.73	94.34	94.74	**100.00**	69.48
**Cons**	**60.91**	**67.71**	68.60	**76.22**	**83.58**	87.50	**91.55**	94.34	92.11	90.48	**72.46**
**Cons-Int**	54.68	64.58	67.77	74.59	80.60	**88.54**	90.14	92.45	92.11	100.00	69.67

See the caption of Table 2 for details. MoCA and MDS-UPDRS part 3 are excluded as they do not contain self-reported items.

**Fig. 1:**
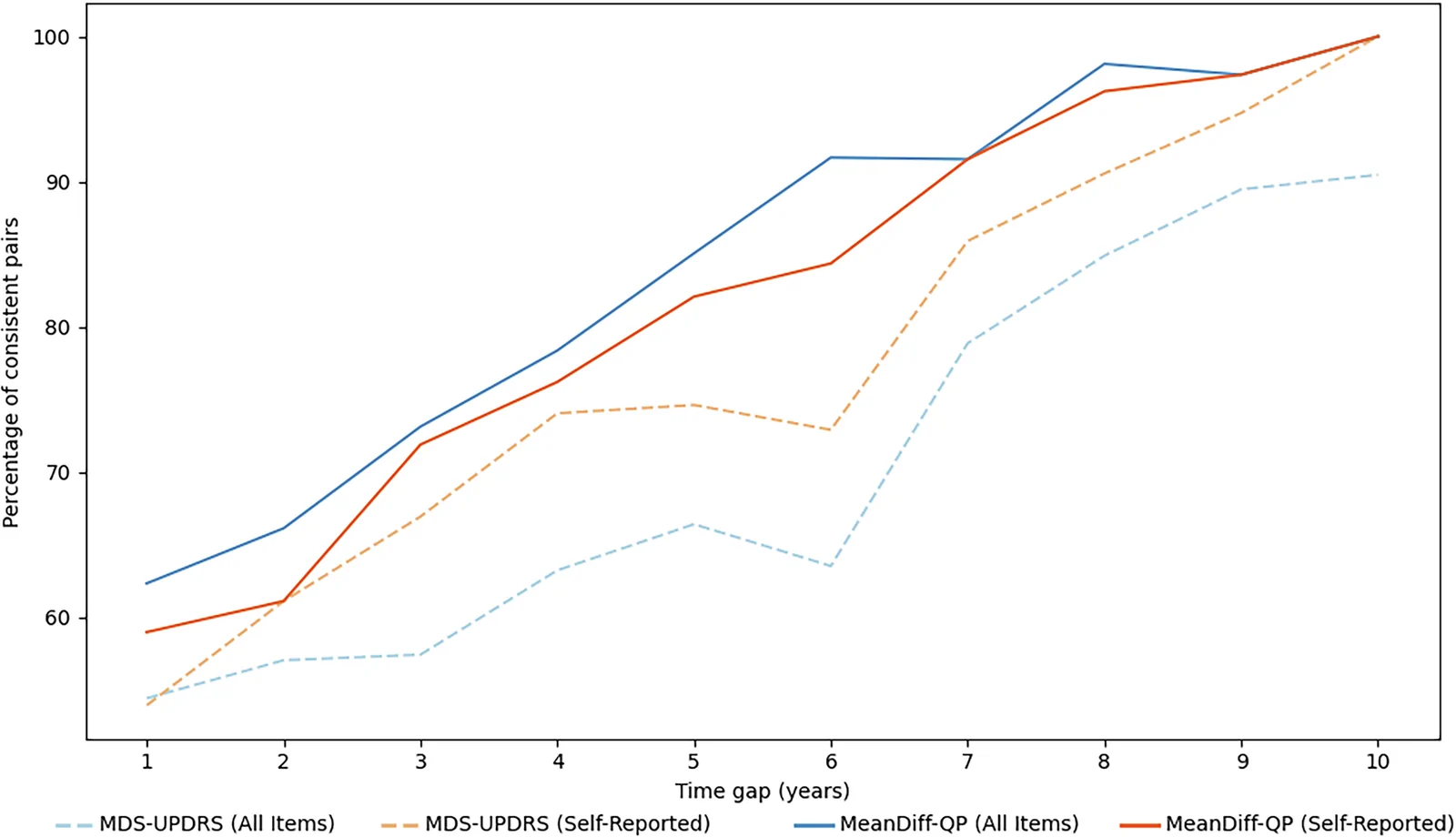
Percentage of consistent pairs for MDS-UPDRS and our MeanDiff-QP scale in various time gaps. MDS-UPDRS: Movement Disorder Society’s Unified Parkinson’s Disease Rating Scale.

Figure 1 compares the performance of MeanDiff-QP and MDS-UPDRS when all items are used and when only self-reported items are used. Remarkably, in both cases our optimized method shows better consistency compared to MDS-UPDRS across all time gaps. Moreover, the self-reported version is almost as good as what we can get with all items.

The new indexes outperformed MDS-UPDRS even when the first visit was included and when patients with dyskinesia were retained in the analysis. This held true both when using all items and when restricted to self-reported items (results not shown).

To ensure that the improved consistency is not a result of a specific train/test split, we repeated the analysis with 20 random splits. The results validated the stability of our findings- see Supplementary Information and Figs. S3, S4.

### Reducing the number of items

**Fig. 2:**
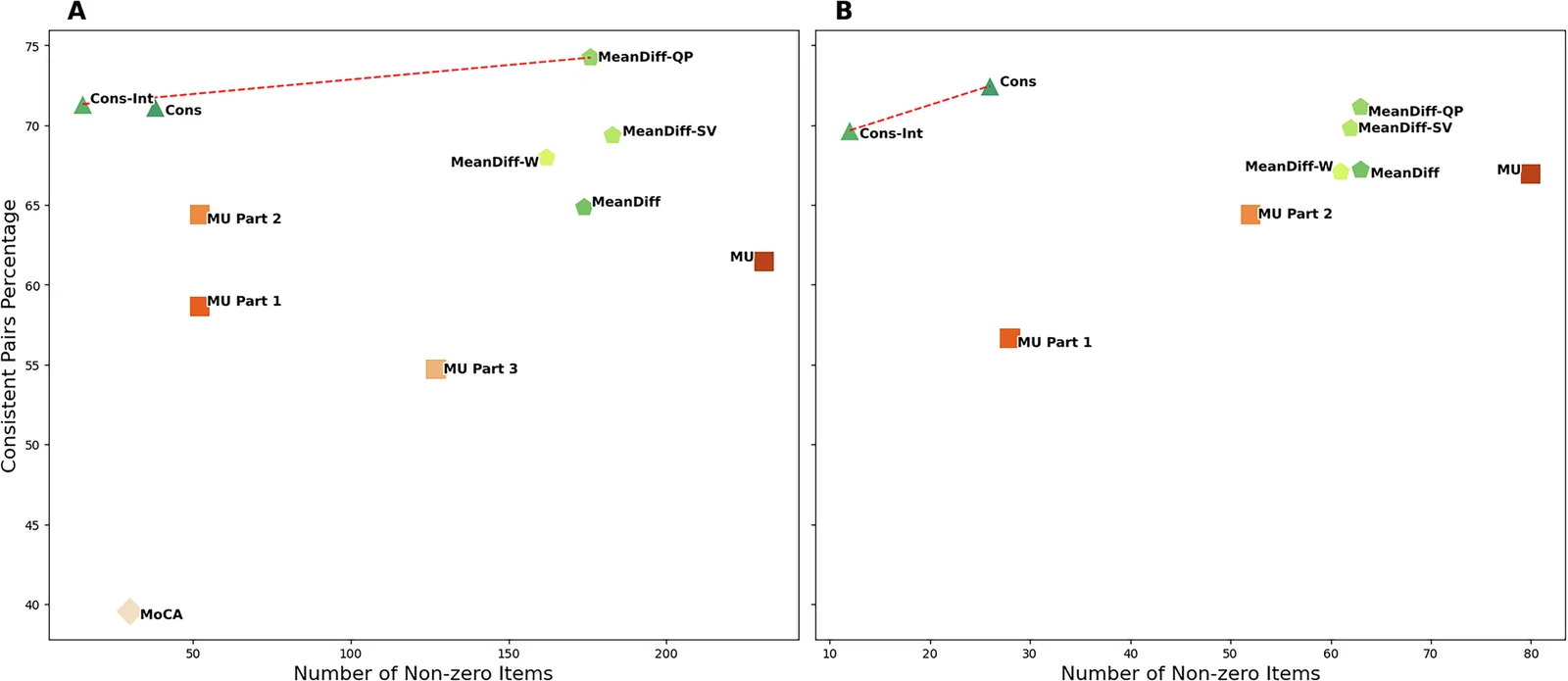
Consistency versus number of items used by each method. Each point represents a method, with the x-axis indicating how many items it includes and the y-axis showing the percentage of visit pairs with increasing scores over time. Methods with the same x-value can be compared by their consistency (higher y is better), while those with the same y-value can be compared by efficiency (lower x is better). An item refers to a single unit increase in a response on the original scale. **A** Performance when using all items. **B** Performance when using only self-reported items. The red lines indicate the Pareto optimal contour. MU Movement Disorder Society’s Unified Parkinson’s Disease Rating Scale (MDS-UPDRS). MoCA Montreal Cognitive Assessment.

Figure 2 shows, for each method, its consistency and the number of non-zero items used. In Fig. 2A all items were considered, and in Fig. 2B only the self-reported items were allowed.

In both cases Cons-Int achieved very good consistency, while using a very small number of items. Table 4 shows the learned weights for that solution using only self-reported items. Remarkably, only eleven questions are used, and in ten of those only one threshold value is needed. In the 11th (Getting out of bed) two thresholds are needed. Put differently, this scale uses only twelve self-reported items yet it outperforms the original 200-item MDS-UPDRS. The only scale to achieve higher consistency is MeanDiff-QP with 176 items. Table S3 gives the weights of Cons-Int when all items are allowed.

**Table 4:** The scale obtained by Cons-Int using only self-reported items

Item	Threshold	Score
1.7 Sleep problems	1	13
1.8 Daytime sleepiness	1	25
1.10 Urinary problems	2	55
1.11 Constipation problems	1	43
1.13 Fatigue	1	30
2.1 Speech	1	44
2.2 Saliva and drooling	1	51
2.9 Turning in bed	1	66
2.11 Getting out of bed	1	52
2.11 Getting out of bed	2	45
2.12 Walking and balance	2	100
2.13 Freezing	1	67

Only the non zero weights are shown. The final index is obtained by summing the scores for all rows in which the item’s value is equal or larger than the threshold.

The learned weights for all methods are provided as supplementary data files. Supplementary Data File 1 contains the weights for scales based on all items. Supplementary Data File 2 contains the weights of scales using only self-reported items.

### Initiation of symptomatic therapy

**Fig. 3:**
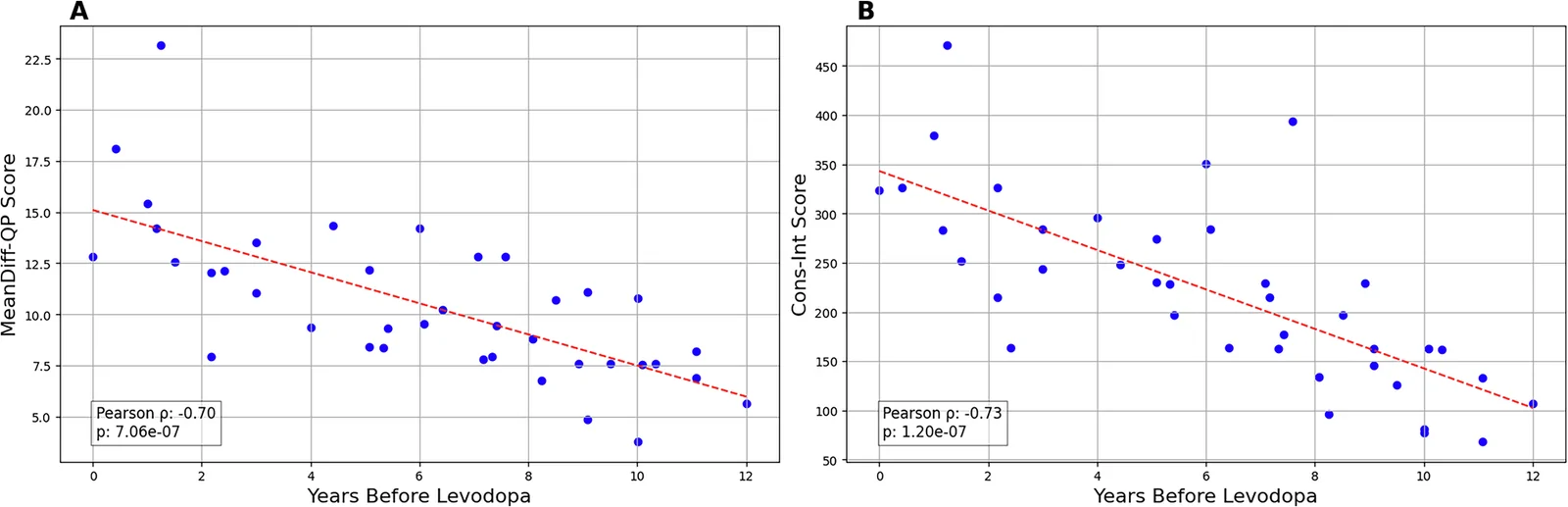
Total scores vs. the number of years prior to initiation of levodopa treatment. **A** MeanDiff-QP using all items **B** Cons-Int using self-reported items only.

Table S6 lists the correlation between each tested method and the time to initiation of levodopa. Indeed, we see highly significant negative correlations between the scores and the time difference. Figure 3 shows the results of the best performing method in terms of the significance of correlation in each scenario: MeanDiff-QP using all items and Cons-Int using just self-reported items.

### Activities of daily living

**Fig. 4:**
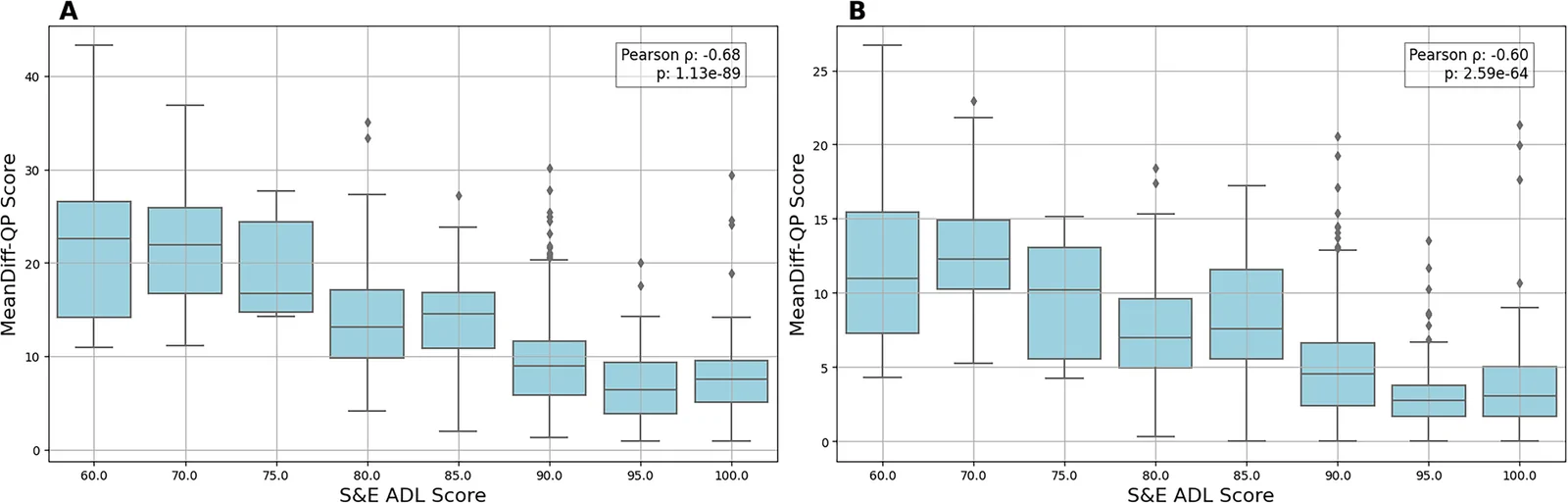
Total scores compared to the S&E ADL scores. **A** MeanDiff-QP results when using all items. **B** MeanDiff-QP results when using only self-reported items.

When testing the scores correlation with the S&E ADL questionnaire^13^, the results of all methods were significant (Table S7). Figure 4 shows the results for MeanDiff-QP, which achieved the highest correlation using all items and the second-best using only self-reported items, surpassed only by MDS-UPDRS Part 2.

Both tests validate the relevance of our suggested scores, showing high correlations to external data that was not a part of the training process. Many methods outperform the original scales, reaching correlations of −0.73 (p = 1.20e-07) with the **Cons-Int** method for time before levodopa treatment and −0.68 (p = 1.13e-89) with **MeanDiff-QP** for S&E ADL.

### Time to milestone

**Fig. 5:**
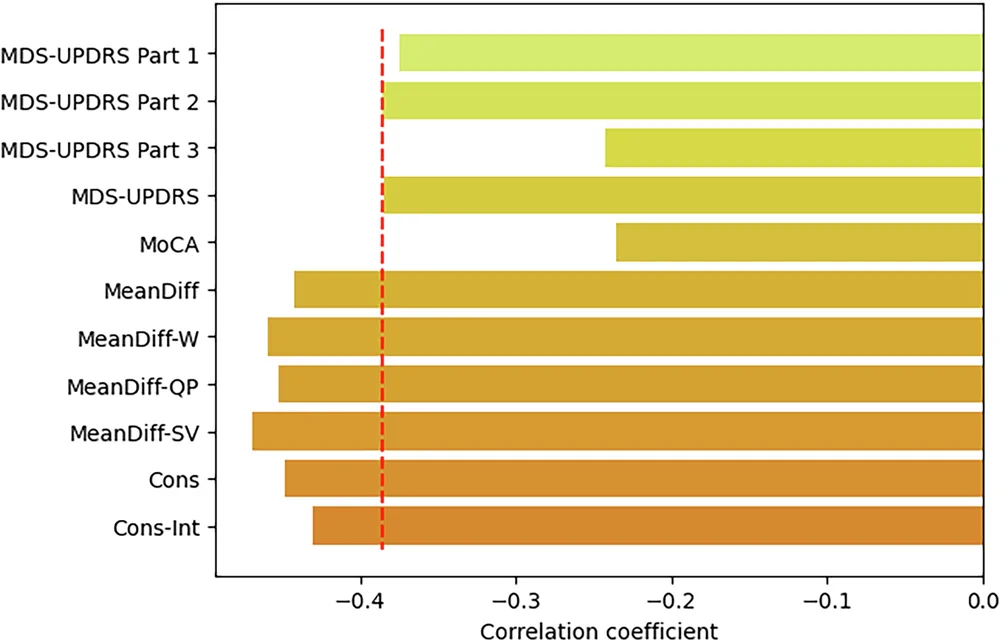
Correlation between the progression index and the time (in months) until the patient reaches at least one of the defined milestones, for each method. All visits after the first visit where a patient reaches a milestone are excluded. The vertical dotted red line indicates the best correlation for a baseline method; our suggested methods exceed this threshold significantly. MDS-UPDRS Movement Disorder Society’s Unified Parkinson’s Disease Rating Scale. MoCA Montreal Cognitive Assessment.

When measuring the correlation between each index and the time to the first milestone as defined in ref. ^14^, all our scales achieved correlation below −0.41, outperforming the MDS-UPDRS. The results for methods using all items can be seen in Fig. 5. The correlation coefficients and *p* values for all methods are available in Table S8.

### ‘OFF’ state visits

Since all our weights were derived from ‘ON’ state data, we evaluated the consistency of the results using these weights on ’OFF’ state visits. Our results show that the proposed methods continued to outperform the baseline MDS-UPDRS. Across all methods, consistency was higher in ‘OFF’ state examinations, suggesting that these assessments better capture the underlying disease state compared to ‘ON’ examinations. The full results for ‘OFF’ visits are described in the Supplementary Information and summarized in Tables S4, S5 and Fig. S2.

### External Validation

The participant characteristics of the BeaT-PD cohort after applying the filtering are described in Table 5.

**Table 5:** BeaT-PD participant characteristics

Characteristic	Mean	Std	Min	Q1	Median	Q3	Max
Age (years)	64.05	11.32	36	57.75	66.5	71	86
Disease Duration (years)	2.82	1.94	1	1	2	4.25	7
MDS-UPDRS Total Score	33.18	16.69	7	21	30	42.5	89
H&Y Stage	1.68	0.57	0	1	2	2	3
MoCA Total Score	24.73	3.49	17	22	25	27.5	30
Gender (M%)	77.22%						
Follow-up Time (years)	3.71	1.6	1	3	3	6	6
Number of Visits	2.54	0.76	2	2	2	3	5

Statistics are shown for the cohort after filtering, consisting of 201 visits of 79 patients. The first five characteristics listed correspond to each patient’s initial visit. Q1: 25th percentile; Q3: 75th percentile.

*MDS-UPDRS* Movement Disorder Society’s Unified Parkinson’s Disease Rating Scale, *MoCA* Montreal Cognitive Assessment, *H&Y* Hoehn and Yahr.

The consistency of all the tested methods on the BeaT-PD cohort is shown in Table 6. Reassuringly, all but one method exceeded the performance of the strongest baseline scale, supporting the robustness of our approach. The improvement of our approach over the best baseline method is statistically significant—see Supplementary Information. Validation results using only self-reported items are available in Table S9.

**Table 6:** External validation results on the Beat-PD cohort

Method	Consistency (%)
**MDS-UPDRS P1**	62.29
**MDS-UPDRS P2**	65.71
**MDS-UPDRS P3**	61.71
**MDS-UPDRS**	65.71
**MoCA**	54.29
**MeanDiff**	69.14
**MeanDiff-W**	70.29
**MeanDiff-QP**	67.43
**MeanDiff-SV**	**71.43**
**Cons**	64.57
**Cons-Int**	67.43

The percentage of consistent pairs of visits for each method on the external validation BeaT-PD dataset, evaluated using the scale derived from the PPMI data. Bold indicates the best performer.

### An online tool

We created an online tool that calculates our progression index using the self-reported answers, available via https://shamir-lab.github.io/MOPS/self_report_short.html. The tool uses the weights of Table 4, normalized so that the range is 0-100.

## Discussion

We introduced a method for optimizing PD progression indexes by reweighting items and increments in the MDS-UPDRS and MoCA scales. The new indexes have higher precision and efficiency, benefiting both patients and clinicians. Our main findings are:

1. Compared to the current approach of merely summing raw item values, our indexes enhance score consistency with disease progression while maintaining a simple “sum-of-items” format.
2. Indexes based solely on self-reported items perform on par with, or in some cases better than, the full MDS-UPDRS scale, including the parts that are clinician-rated.
3. Indexes using only a few items are almost as good as those based on all items.

In particular, eleven self-reported items and twelve weights outperformed the original MDS-UPDRS, which includes 59 items and 236 weights. These findings were corroborated by strong correlations with external progression markers and validated in an external cohort. Our findings support two complementary applications: (1) deriving an optimized composite index while preserving the full MDS-UPDRS for targeted assessments and secondary analyses; and (2) employing a concise, high-signal subset of items to reduce assessment burden and facilitate remote, high-frequency monitoring.

Our research has several implications for clinical practice. First, by removing questions that contribute minimally to tracking PD progression, one can focus on the more meaningful indicators of progression without sacrificing diagnostic or prognostic accuracy. Second, the potential to base progression tracking on properly weighted self-reported items alone enables more frequent as well as remote evaluations, offering patients the flexibility to complete assessments at home, while reducing the burden from clinicians. Importantly, our decision to train only on data of patients in ’ON’ state leads to indexes that are applicable to the real-world daily presentation of patients. Overall, the optimized index could enhance the quality and efficiency of patient care and improve long-term disease management. For transparency and continuity, we recommend reporting both the traditional MDS-UPDRS total score and the optimized index side-by-side whenever feasible. This approach preserves backward compatibility with existing literature while highlighting the progression-relevant signal captured by the new index.

The MoCA score exhibited low consistency according to our analysis, and had a minimal contribution to the indexes, likely due to two factors. Many PD patients, particularly in the early stages, do not experience significant cognitive decline. More importantly, MoCA performance is affected by practice effect, where repeated tests lead to improved scores independent of actual cognitive changes^15^.

It is noteworthy that the score based on part 2 of MDS-UPDRS only outperforms the full MDS-UPDRS score. In particular, it outperforms part 3, which is often regarded as the most clinically relevant and reliable. This can be attributed to the influence of medications, which strongly affect the motor symptoms assessed in part 3. Changes in medication or dosage adjustments frequently lead to lower part 3 scores when patients are in the ON state (as in the dataset used here). Additionally, part 2 assessments, being self-reported, avoid the inter-rater variability that affects part 3, reducing measurement noise and improving consistency. Lastly, while part 3 measures the present state, part 2 items usually ask about the last week, making them less susceptible to symptoms fluctuations. Note, however, that although self-assessments avoid the issue of inter-rater variability that affects part 3, for some purposes differences in the way different patients perceive and report their disturbances may themselves be clinically relevant. Moreover, we are not arguing that interviews are unnecessary to assess PD progression. For instance, interviews may influence subsequent self-reported responses, as clinicians often ask targeted questions that can alter how patients perceive their symptoms and report them in the corresponding MDS-UPDRS items. Accordingly, a possible tiered application of the new index can involve concise, weighted part 2 monitoring for routine follow-up, with clinician-led interviews initiated in response to alerts or diagnostic nuance.

Other approaches were proposed for optimizing the MDS-UPDRS scale. Item Response Theory (IRT)^16^ assumes that each person’s responses are influenced by an underlying trait—in our case, PD severity—and estimates how each item relates to this trait. While IRT has been applied to the MDS-UPDRS^17–22^, it has some limitations. First, the IRT model assumptions are not fulfilled by the MDS-UPDRS^23^. In particular, the assumption that each item is measuring the same trait independently does not hold for the diverse symptoms of PD. Second, IRT does not incentivize sparsity, as it fits the optimal parameters for each item or question separately. Lastly, IRT is primarily designed for cross-sectional data and does not effectively capture changes over time, which are crucial for tracking disease progression. Additionally, recent studies have proposed re-weighting MDS-UPDRS items using partial least squares regression^24,25^. These methods optimized an internal criterion—the mean-to-standard-deviation ratio—which differs from our focus on consistency. Morinan et al.^26^ sought to shorten the scale by selecting a subset of eight items suitable for remote monitoring, optimizing explained variance in the process. Unlike our approach, none of these studies allowed for assigning different weights to individual score increments within the same item.

Since PPMI mostly enrolls patients in an early stage of the disease, our data is biased towards early patients; for example, 92.8% of the exams are of patients with H&Y stage ≤2. Therefore, the utility of our progression scale will be highest for earlier PD patients, and less informative for more advanced patients. While it is mathematically easy to balance the index and adjust the optimization target to give more weight to more severe patients, we decided against such a change for a few reasons. First, a progression index is much more valuable in earlier stages of the disease, since in later, more severe stages it is easier to identify the progression manifested in a wide range of symptoms. Second, giving more weight to patients with higher H&Y will introduce additional noise and bias, as these stages are characterized by specific aspects of PD, and do not capture the full range of symptoms. Moreover, the H&Y staging itself also exhibits moderate inter-rater reliability^27^.

Previous research shows that the tremor items in part 3 contain limited information about the underlying state in PD and do not show worsening over time^28^. Additionally, an IRT scoring of part 3 items gives negative coefficients to the tremor items, claiming they are anti-correlative to the other part 3 items^18^. One contributing factor might be that these items are strongly affected by PD medications like levodopa^29^. Indeed, in our computational approaches these items usually receive little or no weight, supporting the observation that they are poor indicators of PD progression.

Our study explored two ways to rescale the data, with notable difference in their computational hardness. The first method focused on an objective that is slightly different from consistency, but it still often led to well-performing scales. We were able to optimize this objective efficiently using fast algorithms. The second method aimed directly at maximizing consistency, but this made the problem much more challenging to solve (in fact, it is proven to be computationally hard-see Supplementary Information). To tackle it, we used algorithms that can be very slow for large problems. As a result, these algorithms could only find approximately optimal solutions within the available time.

Our study has several limitations. First, we constructed our scales using only data from patients who are drug-naïve or in ON state. This aimed to ensure our results are applicable to patients in their typical daily conditions, where medications are not intentionally withheld. Future studies can use our methodology while focusing on more advanced PD patients who naturally experience frequent OFF-state periods. A more detailed pharmacological profile for each patient—capturing medication types, dosage, and timing—may also allow the model to reweight items dominated by temporary symptomatic relief rather than true disease progression. Second, due to limited computational resources, we split the data into training and test sets but did not allocate a separate validation set for extensive hyperparameter tuning. Instead, for each formulation we tried several parameter values on the training set and took one that performed best. A more systematic approach (e.g., nested cross-validation) using more computation power may lead to better parameter choices and improve the scales. Finally, including prodromal patients can similarly expand the applicability of our approach, enabling earlier and more nuanced detection of progression trajectories.

It is worth noting that the computational approach and methods provided here can lead to improvement in scales of other diseases or conditions. Examples include ADAS-Cog^30^ for Alzheimer’s disease (AD) evaluation, the RENAL nephrology scoring system^31^, the Glasgow Coma Scale^32^, the Barthel Index for activities of daily living^33^, the Mini-Mental State Examination (MMSE) for cognition^34^, the NIH Stroke Scale^35^ and many others. Weighting and optimization approaches different from ours have been applied in other contexts, including Alzheimer’s disease^36,37^, stroke^38^, and the Barthel Index^39–41^. Since such scores are broadly used in healthcare, improving and simplifying them can increase their utility.

## Methods

### Study design and setting

We conducted a **retrospective observational cohort** study using two PD cohorts.

The primary dataset was from PPMI, an international, multi-center cohort with longitudinal assessments (MDS-UPDRS, MoCA and others) at 3/6/12-month intervals; data was downloaded on 2024-08-07. We included PD participants’ ON-state or drug-naïve visits, excluded visits with dyskinesia interference and all prodromal subjects, and removed baseline visits. The final dataset comprised 3295 examinations from 711 patients. All participants enrolled in the PPMI cohort provided written informed consent. The PPMI study received approval from multiple institutional review boards/ethics committees at all participating sites for PPMI. The study adhered to the principles outlined in the Declaration of Helsinki. Additionally, PPMI is registered with ClinicalTrials.gov under the identifier NCT04477785.

The external validation was performed on the BeaT-PD cohort. After analogous filtering (baseline retained to preserve size), the set included 201 visits from 79 patients. For self-report analyses, all relevant samples were included. The BeaT-PD project is an ongoing study conducted in the Laboratory for Early Markers of Neurodegeneration (LEMON) at the Tel Aviv Sourasky Medical Center (TASMC). The ethical committee of TASMC, according to the guidelines of the Helsinki Declaration, approved the study (approval 204-16TLV). All participants provided informed written consent prior to participation.

For weight optimization and testing, PPMI was randomly split 80/20 by patient, so that all visits of the same patient are in the same split. We learned item/threshold weights on training data and evaluated *longitudinal consistency* (proportion of within-patient pairs with higher later scores) in the held-out PPMI test set and in BeaT-PD. Robustness checks included 20 random splits and evaluation on OFF visits.

### Data

We used data from the Parkinson’s Progression Markers Initiative (PPMI)^11^ - an international, multi-center longitudinal study aimed at identifying biomarkers of PD progression. In this study, various assessments including MDS-UPDRS are conducted regularly at intervals of 3, 6, or 12 months to track changes in clinical and cognitive status over time (See Supplementary Information for additional details). While PPMI contains a variety of data types including imaging and genetic data, for constructing the new index we only used MDS-UPDRS^42^ for summarizing patients’ clinical state and MoCA^43^ for their cognition.

### Filtering

The MDS-UPDRS^2^ is a 65-item scale divided into four parts (Part I: non-motor experiences of daily living, II: motor experiences of daily living, III: clinician-rated motor examination, IV: motor complications). As our input, we used the 59 questions in parts I, II and III as well as MoCA. While PPMI contains various types of subjects (Healthy, PD, prodromal PD and other disorders) we focused only on PD patients in this analysis, and in particular excluded prodromal patients. We also removed examinations where the rater noted that dyskinesia interfered with the rating, and examinations with missing data (no imputation was performed).

Since we wanted our tool to be applicable in regular clinical visits, we excluded visits where the PD patients were measured in ’OFF’ state, as this kind of measurement often requires patients to purposely stop taking their medications and experience their symptoms more severely. However, we also validated our learned weights on the ’OFF’ visits.

Finally, we removed the baseline visit of each patient from our analysis, as we suspect the first visit might be biased due to the Hawthorne effect^44^, as the act of joining a clinical trial by itself might create some temporal positive “improvement” in the patient’s state, compared to follow-up visits.

After filtering the data we had a total of 3295 examinations for 711 different patients (averaging in 4.63 exams per patient, with median time difference between adjacent visits of 1 year). All eligible visits were part of the analysis. Note the data is not distributed evenly across PD severity levels, and is heavily biased towards early patients. The participant selection flow diagram is presented in Fig. S1. See supplementary Information for the additional details on the PPMI data.

### Encoding

To make the data canonic and usable for the next step, we transformed it as follows. First, while MDS-UPDRS gives higher scores for more severe patients, the MoCA score decreases with severity from 30 to 0 - patients get full points for correct answers. To have both monotone increasing with severity, we flipped the values of each MoCA item such that the value is the number of points deducted instead of the number of points gained. For the verbal fluency item, we used the exact number of words rather than a binary indicator whether the count reached 11, to allow greater flexibility in determining the weights.

Next, for each question, we assigned a binary variable for each unit increment in the answer. For example, an MDS-UPDRS question that can have an answer between 0 and 4 was transformed into four binary variables *x*_1_, *x*_2_, *x*_3_, *x*_4_, where *x*_*i*_ = 1 if the answer is at least *i*. Hence, the answer 0 is mapped to [0,0,0,0], 1 is mapped to [1,0,0,0], 2 is mapped to [1,1,0,0], 3 is mapped to [1,1,1,0] and 4 is mapped to [1,1,1,1]. This way, for example, the answers to the 59 questions used from the MDS-UPDRS are represented by 236 binary variables. This type of encoding for ordinal data is sometimes referred to as thermometer encoding^45^ or cumulative binary encoding. By giving non-negative weights to items, *w*_1_, *w*_2_, *w*_3_, *w*_4_, the score of a question ∑_*i*_*w*_*i*_ ⋅ *x*_*i*_ is monotone non-decreasing: Higher answers are assigned higher scores. The total weighted sum of all answers in a patient’s visit is called its *progression index*.

### Evaluation

We randomly split the data into 80% training set and 20% test/evaluation set, such that no patient appeared in both train and test sets. The learning of weights was done only on the training set, and evaluated on the test set.

Our primary metric for assessing the optimized weights was the percentage of visit pairs for the same patient (considering all possible pairs, not only contiguous visits) in which the later visit received a higher total score. We call this metric *consistency*. A score with higher consistency is better. We also measured the number of non-zero weights assigned to items. A lower number reflects a simpler scale that is easier to implement.

We also compared the computed progression index against external progression criteria, and tested whether it performs better than the baseline approaches. The first set of criteria were based on data available in PPMI. First, we examined the relationship between a visit’s score and the time elapsed from that visit until the start of levodopa treatment, assuming an effective scale should assign higher scores to patients who are closer to beginning treatment. Second, we checked the scores concordance with the Schwab and England Activities of Daily Living (S&E ADL) scale^13^, expecting a negative correlation between our disease progression score and the ADL score. Lastly, we used the milestones defined by Brumm et al.^14^ and checked how well our progression index correlates with the time it would take a patient to reach the first milestone. We tested 20 out of the 25 milestones defined in ref. ^14^, for which a sufficiently large fraction of the visits had data. We assumed a good index should exhibit a strong negative correlation, so that higher scores are associated with a shorter time to reaching the first milestone.

Finally, we validated the consistency of our weights against an additional, external cohort of PD patients obtained from the BeaT-PD project (204-16TLV)^12^. The BeaT-PD cohort included 300 recently diagnosed patients with PD (mean age at recruitment 61.67 ± 10.34 years with mean disease duration of 2.5 ± 1.1 years) who were clinically and genetically assessed over 5 years. After applying filtering criteria similar to those used for the PPMI dataset, as described in Section 4 - but without removing baseline visits, to preserve dataset size - we retained 79 patients with a total of 201 visits.

For the validation of the self-report index we applied a milder filtering approach, and did not exclude visits based on MDS-UPDRS part 3 criteria (clinical state or dyskinesia interference), as these are not self-reported measures.

### Full index vs self-reported index

We also developed an index that uses only MDS-UPDRS items that are self-reported and do not require a trained rater. This index uses only the items in the patient’s questionnaire (the second half of part I and the entire part II).

### Approaches for weights optimization

We developed a variety of formulations for optimizing the weights in the scale. The first set of approaches seek to maximize objective functions that are similar to—but not identical to—the consistency measure, are justified by a solid rationale, and can be optimized efficiently. Empirically, they can be solved to optimality on our data within a few minutes of computation on a standard laptop. These approaches include:

- **MeanDiff** - maximizing the mean difference between pairs of visits of the same patient, across all patients.
- **MeanDiff-W** (Weighted) - similar to the above, but penalizing more for negative differences, corresponding to pairs of visits for which the score decreased. The objective is to maximize the weighted sum of differences.
- **MeanDiff-QP** (Quadratic Penalty) - similar to the former but introducing quadratic penalty for decreases - thus penalizing larger decreases more heavily. The objective is to maximize the sum of differences while minimizing the penalty.
- **MeanDiff-SV** (Small Variance) - similar to the MeanDiff approach, with an additional penalty factor measuring the variance of score differences between visits. The objective is to maximize the mean difference while minimizing the differences’ variance, incentivizing stable increases.

For each of the approaches above, we also added an optional regularization term for minimizing the number of non-zero weights, incentivizing sparse solutions. This was both a goal by itself (as discussed earlier), and was also beneficial to prevent overfitting the training data. The full definition for each of these approaches can be found in Supplementary Information.

Our second set of approaches aim to optimize consistency. They seek weights that will maximize the number of consistent pairs. We considered two variants of this problem: one where weights can have any real value, and one where only integer weights are allowed. We call these formulations **Cons** and **Cons-Int**, respectively. The full definitions are given in the Supplementary Information. The objective functions in these formulations are not convex, so finding the global optimum is computationally harder. We used algorithms that may take exponential time to reach an optimum. In practice, we limited the runtime to a few hours and settled for the best solution found in that time.

### Implementation details

All computations were conducted on a system with an AMD EPYC 7702 processor, featuring 128 logical CPUs (64 cores, 2 threads per core) at 2.0 GHz. The machine runs on GNU/Linux 4.15.0-65-generic within an NVIDIA DGX Server environment. Solving Integer Programming and Mixed Integer Programming formulations was done using the Gurobi Optimizer^46^.

The first set of weights optimization formulations took each up to 30 min to complete using just a single thread. The second set of formulations, which aimed to maximize consistency, dealt with hard computational problem and thus was solved using all available cores and were each allotted a 24-h time limit. Within this timeframe, an optimal solution could not be reached. However, the bound for the gap between the best solution found and the optimal solution ranged between 14.3 and 38.7% across all formulations. These values represent upper bounds, and the actual gaps are likely much smaller.

## Supplementary information

Supplementary information

Supplementary Data 1

Supplementary Data 2

## Data Availability

Access to the PPMI dataset is publicly available upon request at https://www.ppmi-info.org. The BeaT-PD dataset is available from AM upon reasonable request.

## Code availability

The code developed in this paper is available at https://github.com/Shamir-Lab/MOPS.
