# Supplementary material for: Optimizing Parkinson’s disease progression scales using computational methods
Source: NPJ Parkinsons Dis. 2026 Jan 23;12:46. doi: 10.1038/s41531-026-01259-1 (PMC12910052; doi:10.1038/s41531-026-01259-1)
Supplement: Supplementary file 1 [file 41531_2026_1259_MOESM1_ESM.pdf]

Supplementary Information

A Supplementary text

A.1 Data

Our data (downloaded from PPMI on August 7 2024) contained information for 1,879 PD patients, 2,089 Prodromal patients and 400 healthy controls. In our analysis we only used the PD patients' data. Table S1 summarizes the data available for these 1,879 PD patients. Part 3 has more visits because patients are often measured twice - in ON and in OFF states. Of the 16,715 visits with Part 3 data, 7,139 were in ON state, 5,158 in OFF and 4,418 were of drug naive patients.

Exam	Total Visits	Unique Patients	Repeat Patients
MDS-UPDRS Part 1	12,453	1,492	1,284
MDS-UPDRS Part 2	12,467	1,496	1,284
MDS-UPDRS Part 3	16,715	1,497	1,285
MoCA	6,544	1,709	1,096

Table S1: Statistics on the PD patients in the PPMI. Repeat patients are patients who had more than one visit. MDS-UPDRS: Movement Disorder Society's Unified Parkinson's Disease Rating Scale. MoCA: Montreal Cognitive Assessment.

After removing visits in OFF-state and patients with just a single visit, we were left with 4,919 visits of 823 unique patients that contain all MDS-UPDRS parts and MoCA. Removing the baseline and screening visits of all patients left us with 4,269 visits of 763 unique patients. Finally, we filtered visits where dyskinesia interfered with the rating or where critical values were missing or misaligned. The final dataset used in the analysis consisted of 3,295 visits of 711 unique patients (an average of 4.63 visits per patient). Table S2 presents the number of visits in each severity level in the final dataset, showing the bias towards early patients.

**H&Y Stage**	**0**	**1**	**2**	**3**	**4**	**5**
**Number of Visits**	16	599	2445	192	39	4

Table S2: Number of visits for each H&Y stage. H&Y: Hoehn and Yahr.

A.2 Mathematical formulations of progression index problem

A.2.1 Terminology

We start with some basic definitions needed for formulating our problem.

The questions in the original scale have a range of k values of possible answers (For example, $0, \ldots, 4$ in the MDS-UPDRS). Without loss of generality we renumber them $1, \ldots, k$, where higher numbers indicate more severity. Each such question is translated into $k-1$ binary features called *items*, where item i indicates that the answer to the question is at least i.

In this convention, a *visit* is a binary vector $\mathbf{v} \in \{0,1\}^m$, where for each item i, $v_i = 1$ if and only if this item is true for that visit. We denote the j-th visit of patient p by $\mathbf{v}^p_j$. The sequence of visits of patient p is denoted by $(\mathbf{v}^p_1, \ldots \mathbf{v}^p_{m_p})$, where we assume that for each patient p $m_p \geq 2$, and the visits are numbered in increasing time order. A pair of visits $(\mathbf{v}^p_i, \mathbf{v}^r_j)$ is called *proper* if $p = r$ and $i < j$. In words, the two visits should be for the same patient and they should be ordered chronologically.

The formulation assigns a *weight* $w_i \in \mathbb{R}_+$ to each item i, together forming a *weight vector* $\mathbf{w} \in \mathbb{R}^m_+$. Ensuring that item weights w_i are non-negative and defining the weight of an answer with value j as $w_1 + \ldots + w_j$ guarantees that the answer weights are monotone increasing. For a visit $\mathbf{v}$ and weights $\mathbf{w}$, the *score* of the visit is defined as $\mathbf{w} \cdot \mathbf{v}$.

A *longitudinal dataset* is a collection of sequences of visits, one per patient. Formally $\{(\mathbf{v}^p_1, \ldots \mathbf{v}^p_{m_p})| \; p = 1 \ldots, n\}$. For such dataset, we define S as the set of all proper pairs of visits. In other words, $S = \{(\mathbf{v}^p_i, \mathbf{v}^p_j)|i < j, p = 1, \ldots n\}$.

For a proper pair of visits $(\mathbf{v}^p_i, \mathbf{v}^p_j)$ and weights $\mathbf{w}$, if $\mathbf{w} \cdot \mathbf{v}^p_i < \mathbf{w} \cdot \mathbf{v}^p_j$ we say that the pair's order is *consistent with the weights*, or simply that the pair is *consistent.* Note the strict inequality in the last equation. If we allowed instead $\mathbf{w} \cdot \mathbf{v}^p_i \leq \mathbf{w} \cdot \mathbf{v}^p_j$, then the weight vector $\mathbf{w} = 0^m$ would be a trivial set of weights for which all proper pairs are consistent.

We are now ready to define two basic formulations of our problem.

Maximum consistency: Given a longitudinal dataset, find a weight vector $\mathbf{w}$ that maximizes the number of consistent pairs. In other words,

$$max\ |\{(\mathbf{v}_i^p, \mathbf{v}_j^p)|\ i < j \text{ and } \mathbf{w} \cdot \mathbf{v}_i^p < \mathbf{w} \cdot \mathbf{v}_j^p, p = 1, \ldots, n\}| \tag{1}$$

Maximum weighted difference: Given a longitudinal dataset, find a weight vector $\mathbf{w}$ that maximizes the weighted difference across all proper pairs. In other words,

$$max\ \sum_p \sum_{i<j} (\mathbf{w} \cdot \mathbf{v}_j^p - \mathbf{w} \cdot \mathbf{v}_i^p) \tag{2}$$

We will introduce several variations of these objectives in the sequel, and also consider a secondary objective of **sparsity**, aiming to reduce the number of items with non-zero weights.

A.3 Formulations maximizing the weighted difference

A.3.1 Linear Programming

A basic linear programming formulation of the problem is

$$max\ \sum_p \sum_{i<j} (\mathbf{w} \cdot \mathbf{v}_j^p - \mathbf{w} \cdot \mathbf{v}_i^p) \tag{\textbf{MeanDiff}}$$

$$0 \le w_i \le 1 \ \ i = 1, \ldots m \tag{3}$$

This problem has a closed form solution: The objective is equal to $\mathbf{w} \cdot \sum_p \sum_{i<j} (\mathbf{v}_j^p - \mathbf{v}_i^p)$. Define $\mathbf{d} = \sum_p \sum_{i<j} (\mathbf{v}_j^p - \mathbf{v}_i^p)$. Setting $w_i = 1$ if $d_i > 0$ and zero otherwise is an optimal solution.

The following formulation takes into account also the solution sparsity:

$$max\ \sum_p \sum_{i<j} (\mathbf{w} \cdot \mathbf{v}_j^p - \mathbf{w} \cdot \mathbf{v}_i^p) - \gamma \sum_i w_i \tag{4}$$

$$0 \le w_i \le 1 \ \ i = 1, \ldots m \tag{5}$$

The second term is an L1 regularization of the weights, which incentives sparsity. γ is an hyper-parameter that balances between the weighted difference objective and the aim of minimizing the number of used items. This problem too has a closed form solution, since

the objective can be written as $\sum_k w_k \cdot d_k - \gamma \sum_k w_k = \sum_k w_k(d_k - \gamma)$ so setting $w_k = 1$ if $d_k - \gamma > 0$ and zero otherwise is an optimal solution.

Variable pair scores. A possible generalization of the first term in the objective is by assigning different values to different pairs: $\sum_p \sum_{i<j} q(p,i,j)(\mathbf{w} \cdot \mathbf{v}_j^p - \mathbf{w} \cdot \mathbf{v}_i^p)$. The value $q(p,i,j)$ of the pair can be used to reduce the weight of visit pairs for patients that have a lot of visits. For example, if patient p has t visits, then we can make $q(p,i,j) = \frac{1}{\binom{t}{2}}$ to give each patient equal total weight, or $q(p,i,j) = \frac{t}{\binom{t}{2}}$ to make the total weight proportional to the number of visits (as opposed to t^2). Alternatively, we can assign different weights to different pairs of visits based on their time span, as larger time gaps are expected to more strongly capture changes in disease severity.

Penalizing score drops. This approach is similar to the previous one, but instead of simply maximizing the weighted sum of differences, we would like to punish more heavily inconsistent pairs. We show this for the basic formulation. Denote by S the set of all proper visit pairs, where the elements in S are the triplets (p,i,j) such that i and j are visits of patient p with $j > i$. For each $(p,i,j) \in S$ define nonnegative variables $U_{p,i,j}$ (for up) and $D_{p,i,j}$ (down).

$$\max \quad \sum_p \sum_{i<j} \left(U_{p,i,j} - \delta\, D_{p,i,j}\right) \qquad \textbf{(MeanDiff-W)}$$

$$\mathbf{w} \cdot \mathbf{v}_j^p \;-\; \mathbf{w} \cdot \mathbf{v}_i^p \;=\; U_{p,i,j} - D_{p,i,j} \quad \forall (p,i,j) \in S \tag{6}$$

$$0 \;\leq\; w_i \;\leq\; 1, \quad i = 1, \dots, m \tag{7}$$

$$U_{p,i,j},\; D_{p,i,j} \;\geq 0 \tag{8}$$

$\delta > 1$ is the penalty coefficient for inconsistent pairs.

Claim: Any optimal solution of the problem must satisfy:

(i) If $\mathbf{w} \cdot (\mathbf{v}_j^p - \mathbf{v}_i^p) \geq 0$, then $U_{p,i,j} = \mathbf{w} \cdot (\mathbf{v}_j^p - \mathbf{v}_i^p)$ and $D_{p,i,j} = 0$.

(ii) If $\mathbf{w} \cdot (\mathbf{v}_j^p - \mathbf{v}_i^p) \leq 0$, then $D_{p,i,j} = -\mathbf{w} \cdot (\mathbf{v}_j^p - \mathbf{v}_i^p)$ and $U_{p,i,j} = 0$.

Proof: We prove case (i). The proof of (ii) is analogous. If $\mathbf{w} \cdot (\mathbf{v}_j^p - \mathbf{v}_i^p) = 0$ then by (6) $U_{p,i,j} = D_{p,i,j}$. The contribution of this triplet (p,i,j) to the objective is $D_{p,i,j} - \delta D_{p,i,j}$, which is negative since $\delta > 1$ unless $U_{p,i,j} = D_{p,i,j} = 0$.

If $\mathbf{w} \cdot (\mathbf{v}_j^p - \mathbf{v}_i^p) > 0$, suppose $U_{p,i,j} \neq \mathbf{w} \cdot (\mathbf{v}_j^p - \mathbf{v}_i^p)$. Define $d = U_{p,i,j} - \mathbf{w} \cdot (\mathbf{v}_j^p - \mathbf{v}_i^p)$. To satisfy (6), we get $D_{p,i,j} = d$. $d \geq 0$ due to the non-negativity constraints. Assume by contradiction that $d > 0$. The objective function then changes by:

$$U_{p,i,j} - \delta D_{p,i,j} = \mathbf{w} \cdot (\mathbf{v}_j^p - \mathbf{v}_i^p) + d - \delta d = \mathbf{w} \cdot (\mathbf{v}_j^p - \mathbf{v}_i^p) - (\delta - 1)d \tag{9}$$

Since $\delta > 1$ and $d > 0$, we get a strictly worse objective value than if $d = D_{p,i,j} = 0$, in contradiction to the assignment being optimal. ■

A.3.2 Quadratic Programming

Similarly to the linear programming approach, we can also introduce quadratic terms in the objective - thus formulating a quadratic programming problem.

Squaring the changes. This approach simply squares $U_{p,i,j}$ and $D_{p,i,j}$, thus giving more weight to the big changes compared to the smaller ones:

$$max \sum_p \sum_{i<j} (U_{p,i,j}^2 - \delta D_{p,i,j}^2) \tag{10}$$

$$\mathbf{w} \cdot \mathbf{v}_j^p - \mathbf{w} \cdot \mathbf{v}_i^p = U_{p,i,j} - D_{p,i,j} \quad \forall (p,i,j) \in S \tag{11}$$

$$0 \leq w_i \leq 1 \quad i = 1, \dots m \tag{12}$$

$$U_{p,i,j}, D_{p,i,j} \geq 0 \tag{13}$$

Penalizing drops quadratically. Instead of squaring both terms, here we do so just for the drops - so the loss from a drop is bigger than the gain from an increase of the same size. This steers the solution toward greater consistency. We do it by replacing the objective with:

$$max \sum_p \sum_{i<j} (U_{p,i,j} - \delta D_{p,i,j}^2) \tag{\textbf{MeanDiff-QP}}$$

Mixing linear and quadratic penalties. The caveat of the last approach is that it is tolerant to small decreases. To avoid that, we mix both linear penalty and quadratic penalties for drops, using two coefficients:

$$max \sum_p \sum_{i<j} (U_{p,i,j} - \delta D_{p,i,j} - \delta' D_{p,i,j}^2) \tag{14}$$

In our tests we used version (**MeanDiff-QP**), as we preferred a minimal amount of hyper-parameters.

Reducing variance. Another approach utilizing quadratic programming adds a penalty for the variance of score differences. Denote by $\Delta := \frac{1}{|S|}\sum_p \sum_{i<j}(\mathbf{w}\cdot\mathbf{v}_j^p - \mathbf{w}\cdot\mathbf{v}_i^p)$ the mean difference in the total score between pairs of visits. The new objective function is:

$$max\ [\Delta - \frac{\gamma}{|S|}\sum_p \sum_{i<j}(\mathbf{w}\cdot\mathbf{v}_j^p - \mathbf{w}\cdot\mathbf{v}_i^p - \Delta)^2] \qquad (\textbf{MeanDiff-SV})$$

Where again γ is an hyper-parameter that balances between the weighted difference objective and the objective of the differences being more stable.

A.4 Formulations maximizing consistency

In this section we describe formulations that aim to find integer weights that directly maximize the consistency.

Matrix representation. Recall that S is the set of all proper visit pairs, where the elements in S are the triplets (p, i, j) such that i and j are visits of patient p with $j > i$. Denote $s := |S|$. We define a matrix of differences $\mathbf{A} \in \{-1, 0, 1\}^{s\times m}$, such that for every triplet $S_l = (p, i, j)$ and item $a \in \{1, \ldots, m\}$, we have $\mathbf{A}_{l,a} = (\mathbf{v}_j^p - \mathbf{v}_i^p)_a$. In words, $\mathbf{A}_{l,a}$ is the difference in the value of item a between visits j and i of patient p. Since items are binary $\mathbf{A}$'s entries are 1, 0 or -1.

Integer Programming (IP). We define two boolean vectors of indicators $\mathbf{I}^+, \mathbf{I}^- \in \{0, 1\}^s$, and use the following IP formulation:

$$max \sum_{l=1,\ldots,s} I_l^+ \qquad (\textbf{Cons-Int})$$

$$(\mathbf{Aw})_l \geq \varepsilon - C(1 - I_l^+) \qquad l = 1, \ldots, s \tag{15}$$

$$(\mathbf{Aw})_l \leq C \cdot I_l^+ \qquad l = 1, \ldots, s \tag{16}$$

$$(\mathbf{Aw})_l \leq -\varepsilon + C(1 - I_l^-) \qquad l = 1, \ldots, s \tag{17}$$

$$(\mathbf{Aw})_l \geq -C \cdot I_l^- \qquad l = 1, \ldots, s \tag{18}$$

$$0 \leq w_i \leq B \ \ i = 1, \ldots, m \ \ integer \tag{19}$$

$$0 \leq I_l^+, I_l^- \leq 1 \ \ i = 1, \ldots, s \ \ integer \tag{20}$$

Where $0 < \epsilon \ll 1$ is a sufficiently small constant, B is an upper bound on weight values, and C is a large constant such that $C > B\cdot m+\varepsilon$. We call this problem, which maximizes

consistency and requires weight integrality **Cons-Int**. The version where (19) is changed so that weights w_i can be real valued is called **Cons**.

Lemma: Let $\mathbf{w}, \mathbf{I}^+, \mathbf{I}^-$ be a feasible solution of the problem. Then for every $l = 1, \ldots, s$:

1. $(\mathbf{Aw})_l > 0$ if and only if $I_l^+ = 1$.

2. $(\mathbf{Aw})_l < 0$ if and only if $I_l^- = 1$.

3. $(\mathbf{Aw})_l = 0$ if and only if $I_l^- = I_l^+ = 0$.

Proof: (1) Assume $I_l^+ = 1$. From (15) we get $(\mathbf{Aw})_l \geq \varepsilon - C(1-1) = \varepsilon$. Since $0 < \epsilon < 1$, $(\mathbf{Aw})_l > 0$. In the other direction, assume $(\mathbf{Aw})_l > 0$ and $I_l^+ = 0$. Then from (16) we get $(\mathbf{Aw})_l \leq C \cdot I_l^+ = C \cdot 0 = 0$, a contradiction. (2) Assume $I_l^- = 1$. From (17) we get $(\mathbf{Aw})_l \leq -\varepsilon + C(1-1) = -\varepsilon$. Since $0 < \epsilon < 1$, $(\mathbf{Aw})_l < 0$. In the other direction, assume $(\mathbf{Aw})_l < 0$ and $I_l^- = 0$. Then from (18) we get $(\mathbf{Aw})_l \geq -C \cdot I_l^- = -C \cdot 0 = 0$, a contradiction. (3) follows from (1) and (2). ■

Claim: The solution to Cons-Int maximizes consistency.

Proof: By the lemma, our objective is equivalent to consistency. Denote by $\hat{\mathbf{w}}$ an integer vector of weights $0 \leq \hat{w}_k \leq B$ that achieves optimal consistency. We will show that it corresponds to a feasible solution.

For every consistent pair S_l according to $\hat{\mathbf{w}}$ we assign $I_l^+ = 1$ and $I_l^- = 0$. For every inconsistent pair S_l we assign $I_l^+ = 0$, and assign $I_l^- = 1$ if $(\mathbf{A}\hat{\mathbf{w}})_l < 0$ or $I_l^- = 0$ if $(\mathbf{A}\hat{\mathbf{w}})_l = 0$. We claim $(\hat{\mathbf{w}}, \hat{\mathbf{I}}^+, \hat{\mathbf{I}}^-)$ is a feasible solution. Constraints (19) are satisfied by assumption, and (20) by construction. Assume first S_l is consistent, i.e. $(\mathbf{A}\hat{\mathbf{w}})_l > 0$, $I_l^+ = 1, I_l^- = 0$. The constraint (15) holds since $\hat{\mathbf{w}}$ is an integer vector and $\mathbf{A} \in \{-1, 0, 1\}^{s \times m}$, so $(\mathbf{A}\hat{\mathbf{w}})_l \geq 1 > \varepsilon$. Constraint (16) holds since $(\mathbf{A}\hat{\mathbf{w}})_l \leq mB < C$. Constraint (17) holds since $(\mathbf{A}\hat{\mathbf{w}})_l \leq mB < C - \varepsilon$. Constraint (18) holds since $(\mathbf{A}\hat{\mathbf{w}})_l > 0$.

Assume now S_l is inconsistent and decreasing, i.e. $(\mathbf{A}\hat{\mathbf{w}})_l < 0$, $I_l^+ = 0, I_l^- = 1$. The constraint (15) holds since $(\mathbf{A}\hat{\mathbf{w}})_l \geq -mB > -C + \varepsilon$. (16) holds since $(\mathbf{A}\hat{\mathbf{w}})_l \leq 0$. (17) holds since $(\mathbf{A}\hat{\mathbf{w}})_l < 0$ ($(\mathbf{A}\hat{\mathbf{w}})_l \leq -1$). (18) holds since $(\mathbf{A}\hat{\mathbf{w}})_l \geq -mB > -C + \varepsilon > -C$.

Finally, assume S_l is inconsistent and unchanged, i.e. $(\mathbf{A}\hat{\mathbf{w}})_l = 0$, $I_l^+ = 0, I_l^- = 0$. The constraint (15) holds since $(\mathbf{A}\hat{\mathbf{w}})_l \geq -mB > -C + \varepsilon$. (16) holds since $(\mathbf{A}\hat{\mathbf{w}})_l \leq 0$. (17) holds since $(\mathbf{A}\hat{\mathbf{w}})_l \leq mB < C - \varepsilon$. (18) holds since $(\mathbf{A}\hat{\mathbf{w}})_l \geq 0$. ■

Sparsity. To encourage a sparse solution, we can introduce a regularization term to the objective, as before:

$$max \sum_{l=1,\dots,s} I_l^+ - \gamma \sum_i w_i \tag{21}$$

This drives down the sum of the weights, but not sparsity per se. The following IP formulation achieves this goal. To penalize the number of non-zero weights, we introduce helper boolean variables $\mathbf{z} \in \{0,1\}^m$ and formulate the problem as

$$max \sum_{l=1,\dots,s} I_l^+ - \gamma \sum_i z_i \tag{22}$$

$$(\mathbf{Aw})_l \geq \varepsilon - C(1 - I_l^+) \qquad l = 1,\dots,s \tag{23}$$

$$(\mathbf{Aw})_l \leq C \cdot I_l^+ \qquad l = 1,\dots,s \tag{24}$$

$$(\mathbf{Aw})_l \leq -\varepsilon + C(1 - I_l^-) \qquad l = 1,\dots,s \tag{25}$$

$$(\mathbf{Aw})_l \geq -C \cdot I_l^- \qquad l = 1,\dots,s \tag{26}$$

$$0 \leq w_i \leq B \cdot z_i \;\; i = 1,\dots,m \;\; integer \tag{27}$$

$$0 \leq I_l^+, I_l^- \leq 1 \;\; i = 1,\dots,s \;\; integer \tag{28}$$

$$0 \leq z_i \leq 1 \;\; i = 1,\dots,m \;\; binary \tag{29}$$

Where again $\gamma > 0$ balances between consistency and sparsity.

Claim: An optimal solution of the system must satisfy $\sum_i \mathbf{z}_i = |\{i|\mathbf{w}_i > 0\}|$

Proof: We claim $z_i = 1$ if and only if $w_i > 0$, from which the claim follows. If $w_i > 0$ then it must be that $z_i = 1$, to satisfy 27. If $z_i = 1$ but $w_i = 0$, then assigning $z_i = 0$ will increase the objective value by γ without invalidating any constraint, in contradiction to the solution's optimality. ■

Thus, the penalty term is exactly γ times the number of non-zero weights.

Mixed Integer Programming. We can relax the integrality constraints for the weights w_i, and allow them to have real values instead. This yields a Mixed Integer Programming (MIP) formulation, which can be solved similarly to the previous one. In our tests we tried both approaches, and included a regularization term for sparsity.

An alternative objective. By definition, when optimizing for consistency we do not differentiate between the cases where $\mathbf{w} \cdot (\mathbf{v}_j^p - \mathbf{v}_i^p) = 0$ and $\mathbf{w} \cdot (\mathbf{v}_j^p - \mathbf{v}_i^p) < 0$. However, no change is preferred over negative change when pursuing a monotonic score. This can be

achieved using a similar formulation, by adding a penalty term for the number of strictly negative decreases, with a balance parameter γ:

$$max \sum_{l=1,\dots,s} I_l^+ - \gamma \sum_{l=1,\dots,s} I_l^- \tag{30}$$

We note that a sparsity term can also be added to the objective.

A.5 Hardness of the computational problem

In this section we show that the maximum consistency problem is NP-hard.

First, recall the Partial Maximum Feasible Subsystem problem (Partial Max-FS, also called Constrained Max-FS) [**1**, **2**]. In Partial Max-FS we are given a set of linear inequality constraints, where some of them are called hard constraints and the rest are called soft constraints. We wish to find a largest cardinality subset of the constraints containing all the hard constraints and some of the soft constraints that is feasible. Partial Max-FS is NP-Hard and also hard to approximate efficiently [**3**]. It is also NP-hard if the variables are integer or binary, and if the inequalities are strict ($<$) [**2**].

Observe that the consistency maximization problem is a special case of Partial Max-FS. In our case, the variables are binary, the non-negativity constraints are hard, and set of soft constraints is the set of examination pairs, where for each pair we want the score of the later examination to be higher. The optimal weights are those that maximize the number of constraints that are satisfied.

Theorem: The consistency maximization problem is NP-Hard, even when $B = 1$ (i.e., $\mathbf{w} \in \{0, 1\}^m$).

Proof: Observe first that any matrix $\mathbf{A} \in \{-1, 0, 1\}^{s \times m}$ can be viewed as a matrix of differences of a set of visits as defined in Section A.4. This follows by forming a dataset with s patients where each patient i has exactly two visits, and the coordinate-wise differences between the item values in the two visits match the values in row $\mathbf{A}_{i,\cdot}$. By the observation, we can conveniently discuss the problem in terms of constraints, where the weights are the variables.

We show a reduction from the 3-SAT problem. Given a 3-SAT instance with k variables and n clauses, we construct an instance of the consistency maximization problem as follows:

Variable Constraints: Set $m = 2 \cdot k$, where for each variable x_i, the index $2i-1$ corresponds to the positive literal x_i, and the index $2i$ corresponds to the negation $\neg x_i$. For each variable x_i, introduce two inequalities:

$$w_{2i-1} - w_{2i} \geq 1 \tag{31}$$

$$w_{2i} - w_{2i-1} \geq 1 \tag{32}$$

Observation: If $w_{2i-1} = 1$ and $w_{2i} = 0$, or $w_{2i-1} = 0$ and $w_{2i} = 1$, then exactly one of the inequalities (31) holds. If $w_{2i-1} = w_{2i} = 1$, or $w_{2i-1} = w_{2i} = 0$, then none holds.

Clause Constraints: For each clause C_l in the 3-SAT formula, introduce an inequality corresponding to the literals in the clause. Specifically:

$$w_i + w_j + w_k \geq 1 \tag{33}$$

where i, j, k are the indices corresponding to the literals in the clause.

Clearly, this reduction is polynomial in the size of the 3-SAT input. Note that since the variables $w_i \in \{0, 1\}$ and all the coefficients in (31) and (33) are 0,1, or -1, the constraints ≥ 1 are equivalent to the $\geq \epsilon$ constraints that we had in the Cons-Int formulation.

We claim that the 3-SAT instance is satisfiable if and only if there exists $\mathbf{w} \in \{0, 1\}^m$ that achieves consistency in exactly $n + k$ vectors of differences.

For proof, by the observation, out of each variable-related pair (31) at most one constraint can be satisfied by any assignment. Therefore, the maximal possible number of simultaneously feasible inequalities is $n + k$.

Assume the 3-SAT instance is satisfiable. Then there exists an assignment of the k variables that satisfies all n clauses. Construct the weight vector $\mathbf{w}$ as follows: For each variable x_i: If x_i is True, set $w_{2i-1} = 1$ and $w_{2i} = 0$. If x_i is False, set $w_{2i-1} = 0$ and $w_{2i} = 1$. By the observation, exactly k inequalities corresponding to the variables are consistent. Since all clauses are satisfied by the assignment, each clause inequality has at least one corresponding index in $\mathbf{w}$ set to 1, making all n clause inequalities consistent. Therefore, the total number of satisfied constraints is $n + k$.

Conversely, assume there exists a weight vector $\mathbf{w}$ such that $n + k$ inequalities hold. Since there are k variable-related pairs, at least k of the satisfied inequalities correspond to the variable constraints. By the observation, for each variable x_i, exactly one of the

inequalities (31) holds. If $w_{2i-1} = 1$ and $w_{2i} = 0$, set x_i to be True. If $w_{2i-1} = 0$ and $w_{2i} = 1$, set x_i to be False. Since the total number of satisfied inequalities is $n + k$, it follows that all n clause inequalities hold as well. By construction, a clause vector is consistent if and only if at least one of its corresponding literals is True. Therefore, the assignment satisfies all clauses. ■

B Figures and tables

B.1 Participant selection flow diagram

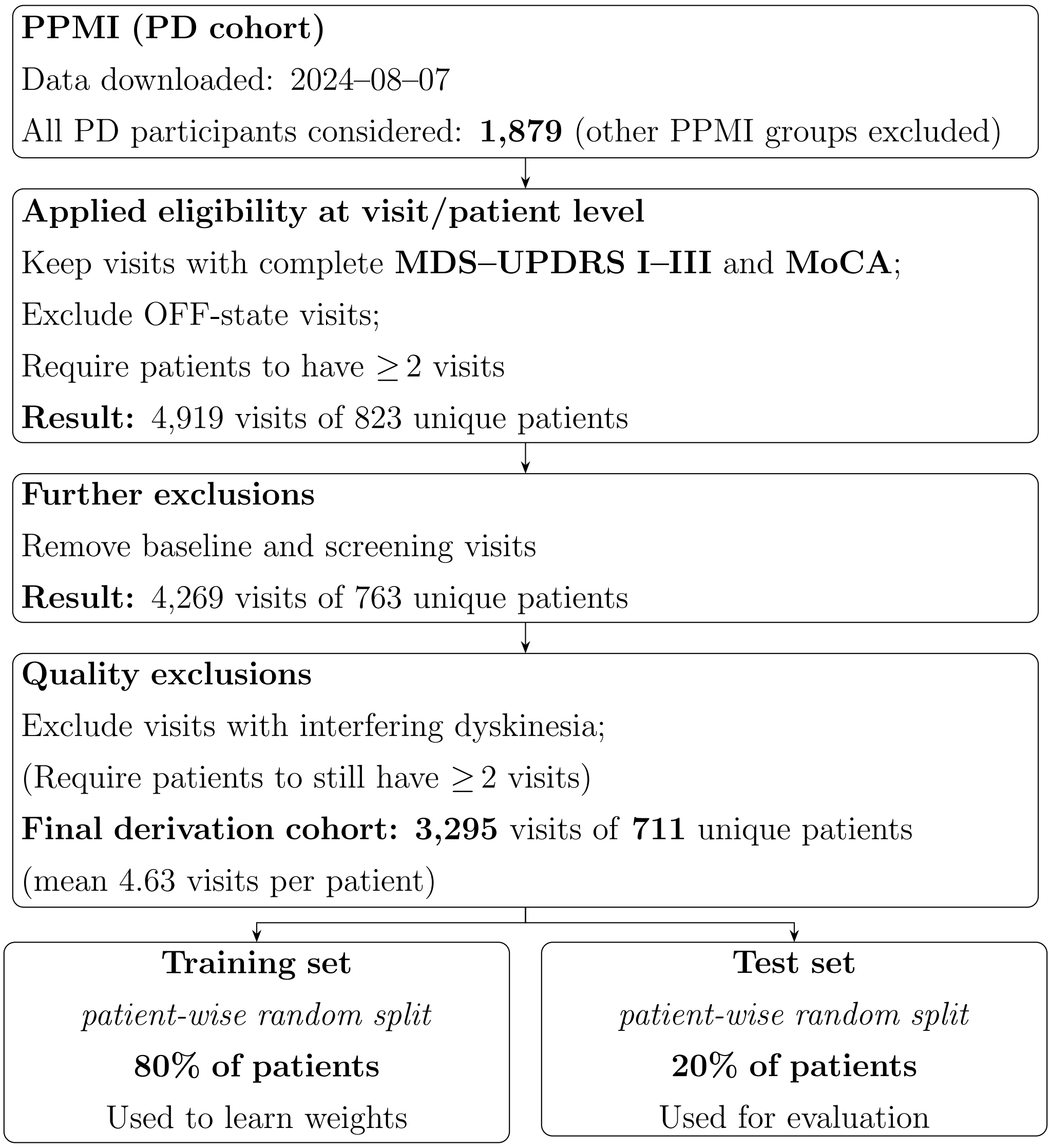

Figure S1: PPMI participant/visit flow. Boxes show successive inclusion/exclusion steps for the full derivation cohort, followed by a random patient-wise 80/20 split into training and evaluation sets. Sensitivity analyses (20 random splits; reanalyses of OFF visits, inclusion of baseline visits and of visits with dyskinesia) were post-hoc checks and are described in the text.

B.2 The weights learned by each approach

Supplementary Data File 1 contains the weights for scales based on all items. Supplementary Data File 2 contains the weights of scales using only self-reported items. For each method, the values were normalized to sum to 100. Note that for MoCA items the values were flipped, so for example a threshold of 1 means 1 point below the maximal possible score.

Item	Threshold	Score
1.1 Cognitive impairment	1	16
1.7 Sleep problems	1	9
1.7 Sleep problems	2	25
2.2 Saliva and drooling	2	14
2.3 Chewing and swallowing	1	10
2.4 Eating tasks	1	13
2.8 Doing hobbies and other activities	1	7
2.9 Turning in bed	1	27
2.12 Walking and balance	2	47
2.13 Freezing	1	35
3.4b Finger tapping - Left hand	1	13
3.7b Toe tapping - Left hand	1	7
3.13 Posture	1	19
3.13 Posture	3	100
MoCA - Clock hands	<1 (fail)	15

Table S3: The scale obtained by Cons-Int when all items can be used. Only non zero weights are shown. The index is obtained by summing the scores for all rows where the item's value is equal or larger than the threshold. MoCA: Montreal Cognitive Assessment.

B.3 Evaluation on 'OFF' visits

While we filtered out visits in 'OFF' state when developing our weights (as discussed in 'method' and 'discussion' sections), we wished to check the consistency of our learned weights on the population of 'OFF' state visits. Tables S4 and S5 show the consistency results for these visits. Remarkably, our learned weights still outperformed the baseline in terms of consistency, even though they were learned on a different medication state.

**Years Gap**	**1**	**2**	**3**	**4**	**5**	**6**	**7**	**8**	**9**	**10**	**All**
**Number of Pairs**	413	328	260	202	163	125	86	73	43	26	1,719
**MDS-UPDRS P1**	48.43	53.66	58.46	64.85	65.03	68.80	68.60	69.86	74.42	73.08	58.87
**MDS-UPDRS P2**	52.78	59.76	64.23	68.81	72.39	76.80	81.40	84.93	95.35	96.15	65.85
**MDS-UPDRS P3**	54.48	61.28	65.38	68.81	72.39	76.00	80.23	87.67	93.02	92.31	66.61
**MDS-UPDRS**	58.11	64.94	71.15	76.24	79.75	85.60	86.05	94.52	93.02	**100.00**	72.02
**MoCA**	38.50	42.07	41.54	41.09	48.47	47.20	36.05	42.47	41.86	46.15	41.77
**MeanDiff**	55.21	64.33	71.54	75.74	82.82	88.00	94.19	94.52	95.35	**100.00**	72.13
**MeanDiff-W**	60.77	68.60	75.38	78.71	83.44	88.80	93.02	**98.63**	**97.67**	**100.00**	75.51
**MeanDiff-QP**	60.29	68.29	**77.69**	82.67	87.73	93.60	94.19	**98.63**	95.35	**100.00**	76.91
**MeanDiff-SV**	61.50	**69.21**	75.38	79.21	83.44	89.60	90.70	97.26	**97.67**	**100.00**	75.74
**Cons**	**62.23**	68.60	**77.69**	**85.64**	**88.96**	**94.40**	**95.35**	**98.63**	**97.67**	**100.00**	**78.07**
**Cons-Int**	56.42	63.72	71.54	82.67	85.28	88.80	**95.35**	93.15	**97.67**	**100.00**	73.47

Table S4: Performance comparison when all MDS-UPDRS and MoCA items are used, evaluated on 'OFF' state visits. The table shows the percentage of consistent pairs of visits for each method, for different time gaps between the visits. Time gaps are rounded to the closest year. The number in bold shows the best performer for each gap. The last column gives the weighted average percentage of consistent pairs. MDS-UPDRS: Movement Disorder Society's Unified Parkinson's Disease Rating Scale. MoCA: Montreal Cognitive Assessment.

Figure S2 shows the results evaluated on 'OFF' state visits for MeanDiff-QP and MDS-UPDRS using all items and using only self-reported items, as a function of time gap between the visits. Note that here the MDS-UPDRS performed better when using all items than when using only self-reported items.

Years Gap	1	2	3	4	5	6	7	8	9	10	All
**Number of Pairs**	413	328	260	202	163	125	86	73	43	26	1,719
**MDS-UPDRS P1**	43.34	50.91	59.62	59.90	58.90	64.00	67.44	75.34	72.09	61.54	55.73
**MDS-UPDRS P2**	52.78	59.76	64.23	68.81	72.39	76.80	81.40	84.93	**95.35**	96.15	65.85
**MDS-UPDRS**	52.06	61.28	67.69	73.76	72.39	74.40	81.40	84.93	93.02	**100.00**	66.90
**MeanDiff**	51.82	61.89	68.85	71.78	74.23	74.40	80.23	87.67	93.02	**100.00**	67.13
**MeanDiff-W**	51.82	60.98	68.08	73.76	75.46	76.00	81.40	86.30	93.02	**100.00**	67.31
**MeanDiff-QP**	57.87	60.98	**73.85**	76.73	79.75	81.60	89.53	87.67	**95.35**	**100.00**	71.32
**MeanDiff-SV**	55.93	65.55	70.00	73.27	75.46	80.00	82.56	90.41	93.02	**100.00**	69.92
**Cons**	**58.60**	**66.16**	70.38	**82.18**	83.44	85.60	**93.02**	90.41	90.70	88.46	**73.24**
**Cons-Int**	52.06	63.11	68.46	80.20	**84.66**	**86.40**	91.86	**91.78**	**95.35**	**100.00**	71.03

Table S5: Performance comparison when only the self-reported items in MDS-UPDRS are used, evaluated on 'OFF' state visits. See the caption of Table S4 for details. MoCA and MDS-UPDRS part 3 are excluded as they do not contain self-reported items.

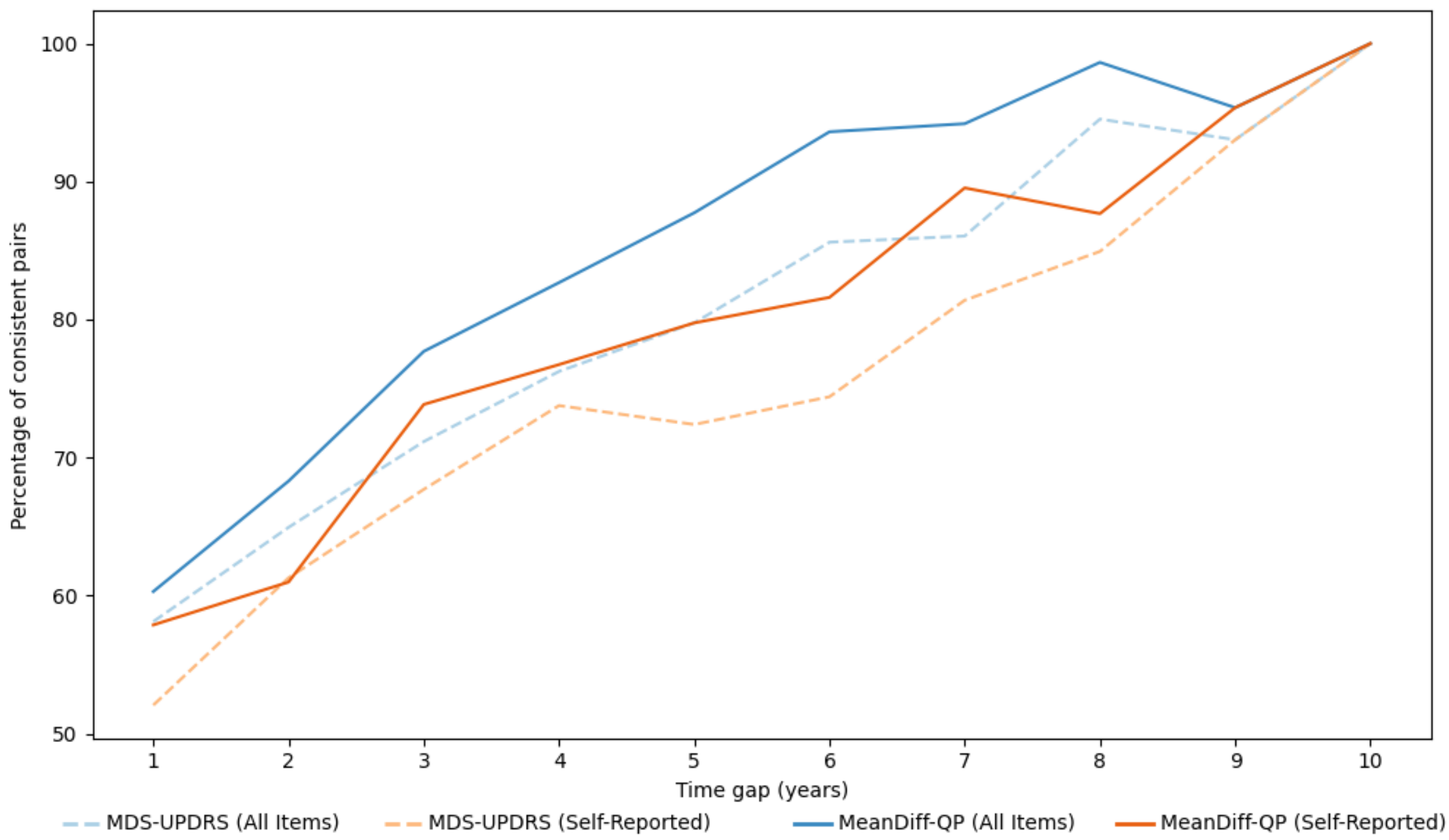

Figure S2: Percentage of consistent pairs evaluated on 'OFF' state visits for MDS-UPDRS and our MeanDiff-QP scale in various time gaps. MDS-UPDRS: Movement Disorder Society's Unified Parkinson's Disease Rating Scale.

B.4 Comparison of the indexes to external scales

Method	All Items	Self-Reported Items
MDS-UPDRS Part 1	-0.31 (p=5.09e-02)	-0.25 (p=1.19e-01)
MDS-UPDRS Part 2	-0.67 (p=3.83e-06)	-0.67 (p=3.83e-06)
MDS-UPDRS Part 3	-0.46 (p=3.44e-03)	NA
MDS-UPDRS	-0.63 (p=2.07e-05)	-0.64 (p=1.15e-05)
MoCA	0.41 (p=1.04e-02)	NA
MeanDiff	-0.62 (p=2.62e-05)	-0.60 (p=4.57e-05)
MeanDiff-W	-0.64 (p=9.98e-06)	-0.61 (p=3.19e-05)
MeanDiff-QP	**-0.70** (p=7.06e-07)	-0.67 (p=3.30e-06)
MeanDiff-SV	-0.69 (p=1.51e-06)	-0.61 (p=4.33e-05)
Cons	-0.62 (p=2.73e-05)	-0.68 (p=1.74e-06)
Cons-Int	-0.60 (p=6.02e-05)	**-0.73** (p=1.20e-07)

Table S6: Correlation between the score of each method and the time to Levodopa. Results are shown for scores that use all items and for scores that use self-reported items only. p-values are calculated using Pearson's ρ. MDS-UPDRS: Movement Disorder Society's Unified Parkinson's Disease Rating Scale. MoCA: Montreal Cognitive Assessment.

B.5 Correlation of the indexes with the time to first milestone

Method	All Items	Self-Reported Items
MDS-UPDRS Part 1	-0.42 (p=3.32e-29)	-0.31 (p=6.62e-16)
MDS-UPDRS Part 2	-0.62 (p=3.51e-69)	**-0.62** (p=3.51e-69)
MDS-UPDRS Part 3	-0.40 (p=2.43e-26)	NA
MDS-UPDRS	-0.59 (p=1.33e-62)	-0.57 (p=2.80e-57)
MoCA	-0.41 (p=2.90e-27)	NA
MeanDiff	-0.65 (p=3.64e-78)	-0.56 (p=8.12e-54)
MeanDiff-W	-0.65 (p=2.55e-78)	-0.56 (p=8.53e-55)
MeanDiff-QP	**-0.68** (p=1.13e-89)	-0.60 (p=2.59e-64)
MeanDiff-SV	-0.62 (p=4.14e-69)	-0.54 (p=1.31e-50)
Cons	-0.64 (p=7.17e-77)	-0.58 (p=1.14e-60)
Cons-Int	-0.57 (p=8.55e-58)	-0.52 (p=2.20e-45)

Table S7: Correlation between the score of each method and S&E ADL. Results are shown for scores that use all items and for scores that use self-reported items only. p-values are calculated using Pearson's ρ. MDS-UPDRS: Movement Disorder Society's Unified Parkinson's Disease Rating Scale. MoCA: Montreal Cognitive Assessment.

B.6 Statistical significance of external validation

To assess whether the improvement of our approach over the baseline was statistically significant in the BeaT-PD validation cohort, we conducted a conservative comparison between MeanDiff-SV and the strongest baseline method, total MDS-UPDRS. Among the 175 pairs of visits, 43 were inconsistent under both methods, 108 were consistent under both, 7 were consistent only under MDS-UPDRS, and 17 were consistent only under MeanDiff-SV. Thus, the proportion of discordant pairs favoring MeanDiff-SV exceeded 0.7. Applying a binomial test to the discordant pairs, with the alternative hypothesis that MeanDiff-SV outperforms MDS-UPDRS, yielded $p = 0.032$.

B.7 Stability of the results

To verify that our outcomes are not biased by the specific randomized train/test split of PPMI data, we ran all MeanDiff variants on 20 random splits of that data and computed the consistency in each case. We excluded from this check Cons and Cons-Int as they

Method	All Items	Self-Reported Items
MDS-UPDRS Part 1	-0.37 (p=3.75e-56)	-0.33 (p=4.54e-44)
MDS-UPDRS Part 2	-0.39 (p=9.49e-60)	-0.39 (p=9.49e-60)
MDS-UPDRS Part 3	-0.24 (p=1.39e-23)	NA
MDS-UPDRS	-0.38 (p=2.00e-59)	-0.41 (p=9.70e-68)
MoCA	-0.24 (p=2.98e-22)	NA
MeanDiff	-0.44 (p=4.69e-80)	**-0.42** (p=3.84e-71)
MeanDiff-W	-0.46 (p=7.03e-87)	**-0.42** (p=5.53e-72)
MeanDiff-QP	-0.45 (p=6.44e-84)	**-0.42** (p=1.94e-71)
MeanDiff-SV	**-0.47** (p=2.97e-91)	**-0.42** (p=2.87e-71)
Cons	-0.45 (p=1.54e-82)	**-0.42** (p=5.42e-73)
Cons-Int	-0.43 (p=1.80e-75)	-0.41 (p=4.55e-67)

Table S8: Correlation between each method's score and the time to first milestone. Results are shown for scores that use all items and for scores that use self-reported items only. p-values are calculated using Pearson's ρ. MDS-UPDRS: Movement Disorder Society's Unified Parkinson's Disease Rating Scale. MoCA: Montreal Cognitive Assessment.

require extensive computation time, as discussed in the methods section.

Figure S3 shows the distribution of the consistency for all methods using all items. Figure S4 shows the distribution of the consistency for the methods using only self-reported items. The new scales demonstrate higher consistency throughout. In fact, in each of the 20 random splits, the MeanDiff variants outperformed all MDS-UPDRS variants. This held true both when all items were included and when the analysis was limited to self-reported items (results not shown).

B.8 External validation using self-reported items

Table S9 shows the consistency results on the external cohort when using only the self-reported items. In this analysis we did not filter by clinical state or presence of dyskinesia, which are relevant to part 3 of MDS-UPDRS. Only one of our suggested methods fell behind the best baseline method. Also, the best performing method using all items, MeanDiff-SV, is ranked second-best in the self-report-only setting.

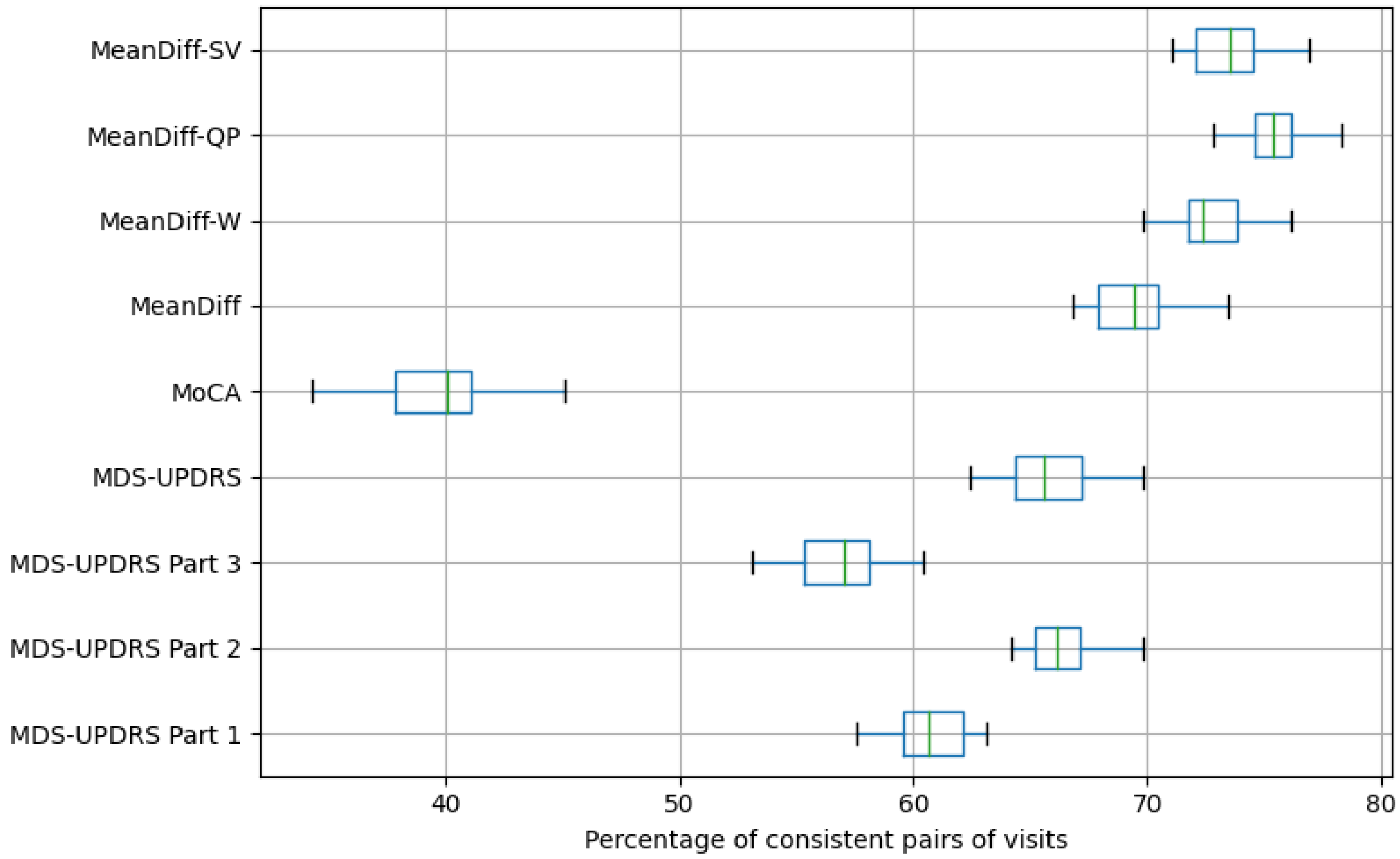

Figure S3: Boxplots of percentage of consistent pairs using all items, across 20 random train/test splits of PPMI data.

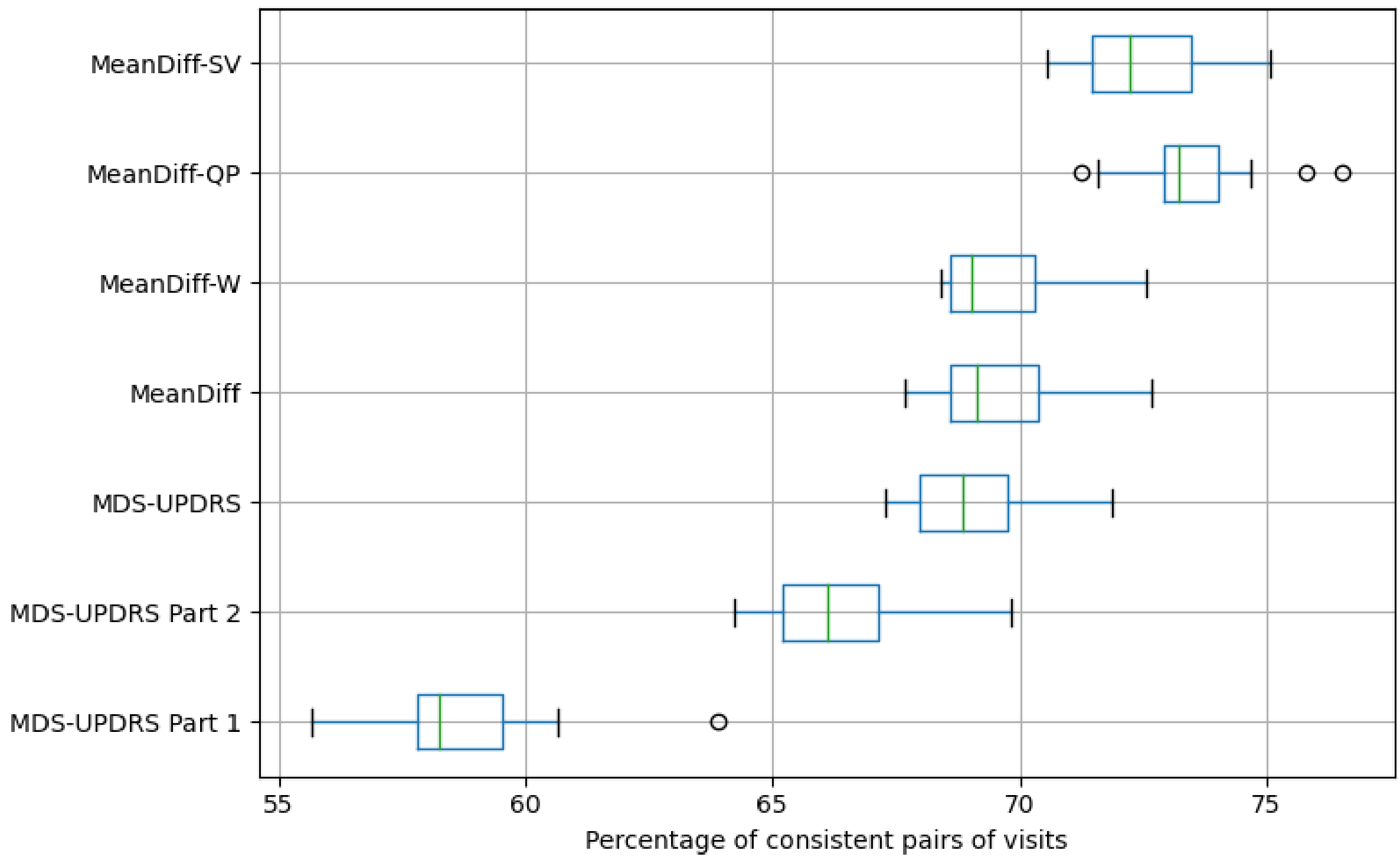

Figure S4: Boxplots of percentage of consistent pairs using only self-reported items, across 20 random train/test splits of PPMI data.

Method	Consistency (%)
**MDS-UPDRS P1**	51.57
**MDS-UPDRS P2**	68.85
**MDS-UPDRS**	68.06
**MeanDiff**	69.63
**MeanDiff-W**	68.85
**MeanDiff-QP**	70.94
**MeanDiff-SV**	71.99
**Cons**	**74.61**
**Cons-Int**	66.23

Table S9: Percentage of consistent visit pairs for each method on the external validation dataset, evaluated with PPMI-derived weights based solely on self-reported items. MDS-UPDRS: Movement Disorder Society's Unified Parkinson's Disease Rating Scale. MoCA: Montreal Cognitive Assessment.

C Supplementary data file captions

Supplementary Data File 1. The weights for scales based on all items. Rows 3–233 represent MDS-UPDRS items (numbered as in the original scale, with separate rows for item thresholds), and rows 234–306 represent MoCA items. For MoCA verbal fluency, the exact word count was used (see Methods). Values were rounded to five decimals, with smaller values set to zero. Columns C–H correspond to the different scales. The final score is computed by summing, for each item with score k, that item's weights for thresholds $\leq k$.

Supplementary Data File 2. The weights for scales based on self reported items. Rows 3–82 represent MDS-UPDRS items (numbered as in the original scale, with separate rows for item thresholds). Values were rounded to five decimals, with smaller values set to zero. Columns C–H correspond to the different scales. The final score is computed by summing, for each item with score k, that item's weights for thresholds $\leq k$.

References

[**1**] Zhaohui Fu and Sharad Malik. "On Solving the Partial MAX-SAT Problem". In: *Theory and Applications of Satisfiability Testing – SAT 2006*. Ed. by Armin Biere and Carla P. Gomes. Vol. 4121. Lecture Notes in Computer Science. Berlin, Heidelberg: Springer Berlin Heidelberg, 2006, pp. 252–265.

[**2**] Edoardo Amaldi and Viggo Kann. "The complexity and approximability of finding maximum feasible subsystems of linear relations". In: *Theoretical Computer Science* 147.1 (1995), pp. 181–210.

[**3**] Edoardo Amaldi, Marc Pfetsch, and Leslie Trotter. "On the Maximum Feasible Subsystem Problem, IISs and IIS-Hypergraphs". In: *Mathematical Programming* 95 (Dec. 2002).
